# sPyNNaker: A Software Package for Running PyNN Simulations on SpiNNaker

**DOI:** 10.3389/fnins.2018.00816

**Published:** 2018-11-20

**Authors:** Oliver Rhodes, Petruţ A. Bogdan, Christian Brenninkmeijer, Simon Davidson, Donal Fellows, Andrew Gait, David R. Lester, Mantas Mikaitis, Luis A. Plana, Andrew G. D. Rowley, Alan B. Stokes, Steve B. Furber

**Affiliations:** Advanced Processor Technologies Group, School of Computer Science University of Manchester, Manchester, United Kingdom

**Keywords:** neuromorphic, PyNN, SpiNNaker machine, spiking neural network (SNN), realtime

## Abstract

This work presents sPyNNaker 4.0.0, the latest version of the software package for simulating PyNN-defined spiking neural networks (SNNs) on the SpiNNaker neuromorphic platform. Operations underpinning realtime SNN execution are presented, including an event-based operating system facilitating efficient time-driven neuron state updates and pipelined event-driven spike processing. Preprocessing, realtime execution, and neuron/synapse model implementations are discussed, all in the context of a simple example SNN. Simulation results are demonstrated, together with performance profiling providing insights into how software interacts with the underlying hardware to achieve realtime execution. System performance is shown to be within a factor of 2 of the original design target of 10,000 synaptic events per millisecond, however SNN topology is shown to influence performance considerably. A cost model is therefore developed characterizing the effect of network connectivity and SNN partitioning. This model enables users to estimate SNN simulation performance, allows the SpiNNaker team to make predictions on the impact of performance improvements, and helps demonstrate the continued potential of the SpiNNaker neuromorphic hardware.

## 1. Introduction

A platform enabling efficient simulation of large-scale networks of spiking neurons (distinct from artificial neural networks, as used in the deep learning community) has the ability to provide advances in multiple areas of research. These include: exploration of scale within the field of computational neuroscience; understanding of brain-like information processing and how it can be harnessed in traditional computational problems; and the ability to embed brain-like systems in physical applications, e.g., facilitating efficient realtime processing in robots such as controllable prosthetics and driver-less vehicles. The benefits of using bespoke hardware to address such problems are well known, and have led to development of the field of neuromorphic engineering (Indiveri and Horiuchi, 2011). Multiple platforms now exist capable of high performance simulation of large numbers of neurons and synapses, often at speeds greatly exceeding biological realtime. Example systems include the Stanford NeuroGrid (Benjamin et al., 2014), IBM TrueNorth (Akopyan et al., 2015), and the BrainScaleS platform (Schemmel et al., 2010) from Heidelberg University; with many bespoke chip-level systems also being developed (Qiao et al., 2015). These systems share a common feature that they offer predefined simulation capability through neurons modeled in analog hardware or digital hardware with fixed software. However, a number of neuromorphic systems instead focus on delivering a flexible platform offering researchers the opportunity to reconfigure simulation functionality to meet their problem requirements. Examples include the Intel Loihi (Davies et al., 2018) system, which has configurable features such as a synaptic plasticity engine for learning; and the SpiNNaker system, which is comprised of fully-programmable ARM cores (Furber, 2016). While this flexible nature compromises these platforms relative to other neuromorphic systems in terms of absolute energy consumption or processing speed, it makes them valuable research tools enabling exploration of new and emerging concepts in computational neuroscience and beyond.

For example, this flexibility has led to the SpiNNaker platform executing large-scale networks of neurons (Sen-Bhattacharya et al., 2017; van Albada et al., 2018), and performing analysis of cognitive tasks such as action selection (Sen-Bhattacharya et al., 2018). Applications of learning in SNNs have also been investigated, such as the study of learning based on Bayesian inference in Knight et al. (2016), and reinforcement learning in Mikaitis et al. (2018). Outside the field of computational neuroscience, SpiNNaker has also been applied to constraint satisfaction problems, e.g., solving computationally hard Sudoku or map color problems in Fonseca Guerra and Furber (2017). In addition to these processing applications, the embodiment of SpiNNaker systems in multiple physical autonomous robots has also been demonstrated, with perhaps the most impressive application being the combination of musculoskeletal robotic hardware with neural control systems implemented on SpiNNaker (Christoph et al., 2016). However, despite these advances, significant challenges are yet to be faced across all of the aforementioned fields. Developing understanding of memory, action selection, and attention will all be crucial in order to take the next steps in understanding of both the brain and brain-inspired systems. The SpiNNaker approach of using programmable ARM cores to simulate neurons and synapses enables upgrading and expansion of system functionality hand-in-hand with developments across the fields of theoretical neuroscience, parallel computing, and robotics. This not only facilitates research in these fields, but provides valuable insights into the design of higher performing, but more “rigid,” hardware-based neuromorphic solutions. However, the creation of such software is unlike that for most other computing machinery (Furber et al., 2013; Brown et al., 2015; Lin et al., 2018); and while development of the SpiNNaker hardware has been well documented (Furber et al., 2013, 2014; Painkras et al., 2013), the software has until now been given less focus.

This paper gives a complete overview of the current state-of-the art in SpiNNaker software pertaining to modeling spiking neural networks. As such, it will focus on describing the software within the sPyNNaker repository^1^, including the internal operations used to simulate networks of neurons, use of hardware resources, and user interaction. This description will assume the existence of SpiNNTools, the low-level graph-based software for managing execution of general parallel software on the SpiNNaker machine, as described in Rowley et al. (2018). While versions of SpiNNaker software have in part been published previously (Jin et al., 2010; Sharp et al., 2011, 2012; Davies et al., 2012), this work contributes a comprehensive overview of the underlying operations and associated hardware interactions which have guided API design. It is hoped this will provide insight to users of the system, and hence improve performance when extending the software framework for future neural applications. As background for the remainder of the paper, this introduction is followed by a high-level overview of both the SpiNNaker hardware, and the PyNN interface for defining networks of spiking neurons. A methods and implementation section follows, providing an in-depth look at the sPyNNaker API developed by the SpiNNaker software team based at the University of Manchester, UK. Results exhibiting performance profiling are then provided. Finally, a discussion section completes the paper, together with suggestions for the future direction of the API.

## 2. Background

### 2.1. The spiNNaker platform—hardware to simulate 1 billion neurons

The SpiNNaker hardware was designed to simulate in realtime networks of spiking neurons, with neurons and synapses modeled in software. The machine targets large-scale networks containing approximately 10^9^ neurons and 10^12^ synapses, giving a 10^3^ mean fan in/out per neuron (Furber et al., 2013), and was scoped to handle mean firing rates of 10Hz (with peak rates of 100Hz). The machine was sized according to the computational power required to simulate synapses, as these typically dominate neural network processing. Assuming processing of a single synaptic event requires 20 instructions (where a synaptic event is defined as a single neuron receiving a single spike), the machine was sized to provide 2 × 10^8^MIPS to handle 10^13^ synaptic events per second. For simplicity and cost reasons, off-the-shelf components were used, specifically the ARM9 core for processing. At 200MHz clock speed, 10^6^ ARM9 cores are required for synaptic processing, and it follows that each ARM core should simultaneously model 10^3^ of the 10^9^ neurons. The remainder of this section gives an overview of the subsequent SpiNNaker chip, including details on chip layout and features pertinent to neural simulation.

**Figure 1:**
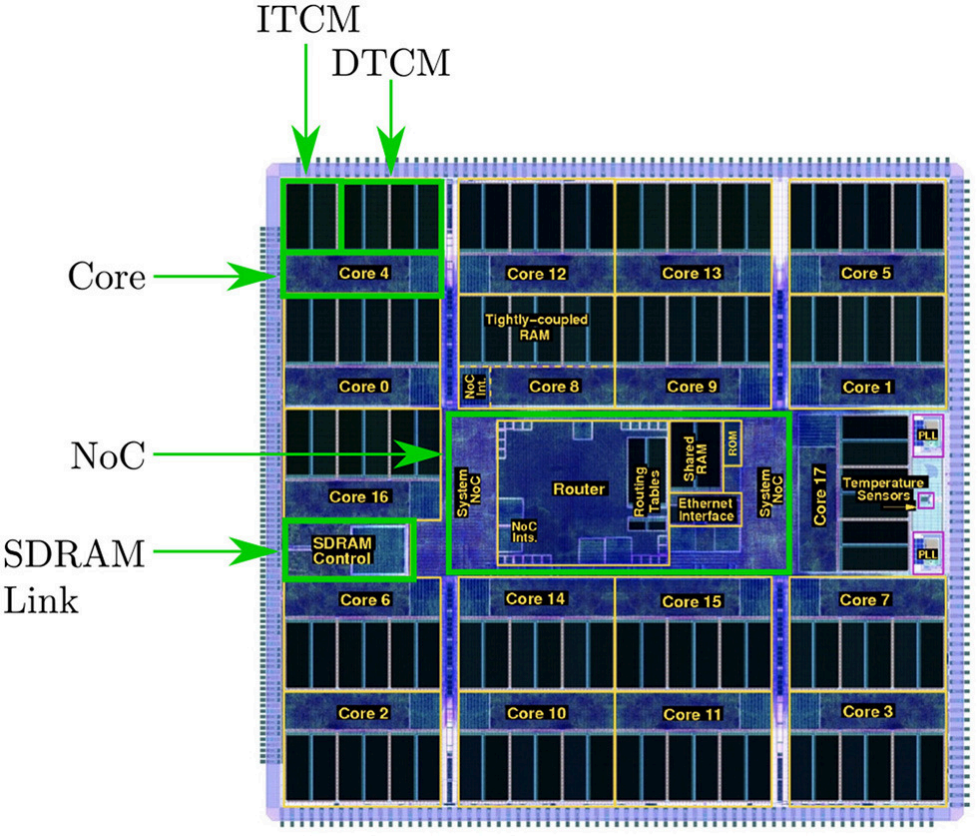
The SpiNNaker Chip, containing: 18 cores, each with local instruction and data memory; network on chip; and SDRAM controller providing access to 128MB of chip-level shared memory.

The SpiNNaker chip houses 18 cores, together with network on chip (NoC) and an external RAM controller—as shown in Figure 1. Each core contains: an ARM968 (ARM, 2004), direct memory access (DMA) controller, communications controller, network interface controller, and other peripherals including the timer. Each core operates at 200MHz clock speed, and typically runs an application simulating a group of neurons. Each core has 96kB of tightly coupled memory (TCM), which to avoid contention is split: 32kB for instructions (ITCM) and 64kB for data (DTCM). Application code is compiled into an ARM968 executable and loaded to ITCM, while DTCM contains application data including heap, stack and other read/write and zero initialized data. Each chip has an additional 128MB of shared memory (SDRAM), directly accessible by all cores on the chip. Access times to these different memories vary considerably, and are therefore a key consideration when designing SpiNNaker software applications.

- Access to DTCM is fast at ≈5ns/word (where unless otherwise stated a word is 32bits), but is limited to only the local core.
- SDRAM can be accessed via bridge, however this is relatively slow at >100ns/word, and subject to contention with other cores.
- A direct memory access (DMA) controller is therefore provided for each core, enabling efficient bulk transfer of data from SDRAM to core DTCM. Setup of the DMA incurs a fixed overhead, however data is then transferred at ≈10ns/word, independently from the processor.

A single DMA request can transfer up to 64kB, but is broken down into smaller data bursts to facilitate transfer. This burst size is typically set at 16 double words, with a small time penalty incurred during setup of each burst. DMA requests are subject to contention at the SDRAM controller, however each DMA controller can submit only a single burst request at a time, introducing a fair queuing policy between cores. Note also that the requests themselves are subject to contention, which can lead to latency in initiation of DMA operations. Despite this contention between DMA requests, the SDRAM controller bandwidth has the capacity to sustain DMA transfers to multiple cores simultaneously, both reading and writing data to and from SDRAM. However, when three or more cores read, and two or more cores write, performance becomes bandwidth limited—with maximum bandwidth reported as 900MBs^−1^ (Painkras et al., 2013; Sharp and Furber, 2013).

Individual SpiNNaker chips are assembled onto boards in a two-dimensional mesh, with common board sizes containing 4 and 48 chips. Multiple boards can then be connected to create a SpiNNaker “machine.” Cores operate in a globally asynchronous locally synchronous (GALS) manner, and communicate through small messages or packets sent via the NoC and SpiNNaker router (Navaridas et al., 2015). The router allows transmission of packets to any subset of the cores on a chip, and to a subset of the 6 off-chip links (enabling chip-to-chip transmission, and hence routing of packets to any core on the machine). The neural applications presented herein use multicast packets, designed to be transmitted from a source to multiple targets simultaneously. A multicast packet contains an 8-bit control byte used by the system, and a 32-bit key used to route the packet. This key is looked up within a table of entries, each of which indicates which of the matching cores and/or links packets should be sent to. This multicast behavior allows a core to send a single message targeting multiple destinations, without having to send an individual message to each of them. It also allows fire-and-forget sending of packets, removing the need for network level interlocking between source and destination. The resulting source-directed routing architecture enables highly efficient message distribution compared to traditional network architectures.

SpiNNaker software applications are typically written in C, and compiled into ARM executable code for maximum execution speed. When designing applications which solve systems of equations, consideration must be given to the impact of precision on results and their numerical stability. The SpiNNaker ARM968 has no hardware floating-point support, and software-implemented floating-point operations are costly in terms of both ITCM and execution time. Fixed point arithmetic is therefore the preferred data representation when solving systems of equations governing neural dynamics. While creation of custom fixed point datatypes is possible, and potentially achieves optimal performance (Jin et al., 2008), the ISO/IEC TR 18037:2008^2^ standard is recommended and used throughout unless otherwise stated. This provides types and operators similar to those defining standard floating point operations, improving ease of reading and code development for non-specialist ARM968 programmers. This standard has been implemented in the GCC toolchain for the ARM target, with version 5.4 the recommended compiler for sPyNNaker 4.0.0. Unless otherwise stated, variables in this work will be defined according to the ISO standard *accum* type: a signed 16-integer and 15-fractional bit fixed-point number, as discussed by Hopkins and Furber (2015). Variables typed as *accum* have an absolute lower limit of 0.000030517578125 (below which underflow occurs and 0 will be returned), and absolute upper limit of 65535.999969482421875 (above which it will overflow and wrap). Division is expensive due to a lack of hardware support, and should be replaced with multiplication by the reciprocal when developing performance-critical applications. An extension to the fixed-point standard implements exponential and logarithm functions, however they are costly to evaluate and hence should be precalculated on host when used as constants to optimize performance.

### 2.2. Pynn

PyNN is a Python interface to define SNN simulations for a range of simulator back-ends (Davison et al., 2008). It allows users to specify an SNN simulation via a Python script once, and have it executed on any or all of the supported back-ends including NEST (Gewaltig and Diesmann, 2007), NEURON (Carnevale and Hines, 2006), and Brian (Goodman and Brette, 2009). This encourages standardization of simulators, reproducibility of results, and increases productivity of neural network modelers through code sharing and reuse, and by providing a foundation for simulator-agnostic post-processing, visualization and data-management tools.

PyNN has continued development as part of the European Flagship Human Brain Project (Amunts et al., 2016), and has hence been adopted as a modeling language by a number of partners including SpiNNaker. It provides a structured interface for the definition of neurons, synapses, and input sources, giving users the flexibility to build a range of network topologies. Models typically consist of single-compartment point neurons, grouped together in “populations.” These populations are then linked with “projections,” representing the synaptic connections between the axons of neurons in a source population, and the dendrites of neurons in a target population. Once defined, a number of simulation controls are used to execute the model for a given time period, with the option to update parameters and initialize state variables between runs. On simulation completion data can be extracted for post-processing and future reference. Neuron variables such as spike trains, total synaptic conductances, and neuron membrane potential are accessible from population objects; while synaptic weights and delays are extracted from projections. This data can be subsequently saved, or visualized using the built-in plotting functionality.

Example PyNN commands for the generation of populations and projections are detailed in **Code 1**. Here the sPyNNaker version of the simulator is imported as *sim*, and subsequently used to construct and execute a simulation. A population of 250 Poisson source neurons is created with label “poisson_source,” and provides 50Hz input to the network for 5s. A second population of 500 integrate and fire neurons is then created and labeled as “excitatory_pop.” Excitatory connections are made between “poisson_source” and “excitatory_pop” with a 20% probability of connection, each with a weight of 0.06nA and delays specified via a probability distribution. Data recording is then enabled for “excitatory_pop,” and the simulation executed for 5s. Finally, the “excitatory_pop” spike history data is extracted from the simulator.


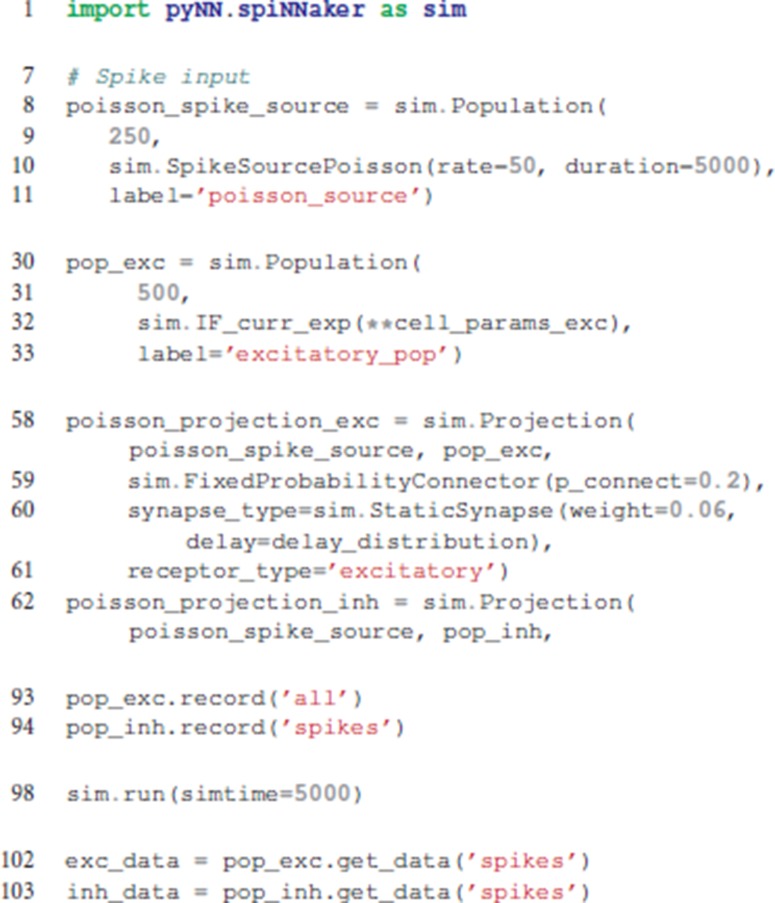


The job of a PyNN simulator is therefore to provide a backend-specific implementation of the PyNN language, enabling execution of simulations defined in model scripts such as Code S1. The PyNN implementation for SpiNNaker hardware (s-PyNN-aker) is detailed in section 3, and is the major contribution of this work. It covers the process of converting a PyNN script into a form suitable for execution on a SpiNNaker machine, and the low-level execution on the machine itself.

## 3. sPyNNaker: methods and implementation

**Figure 2:**
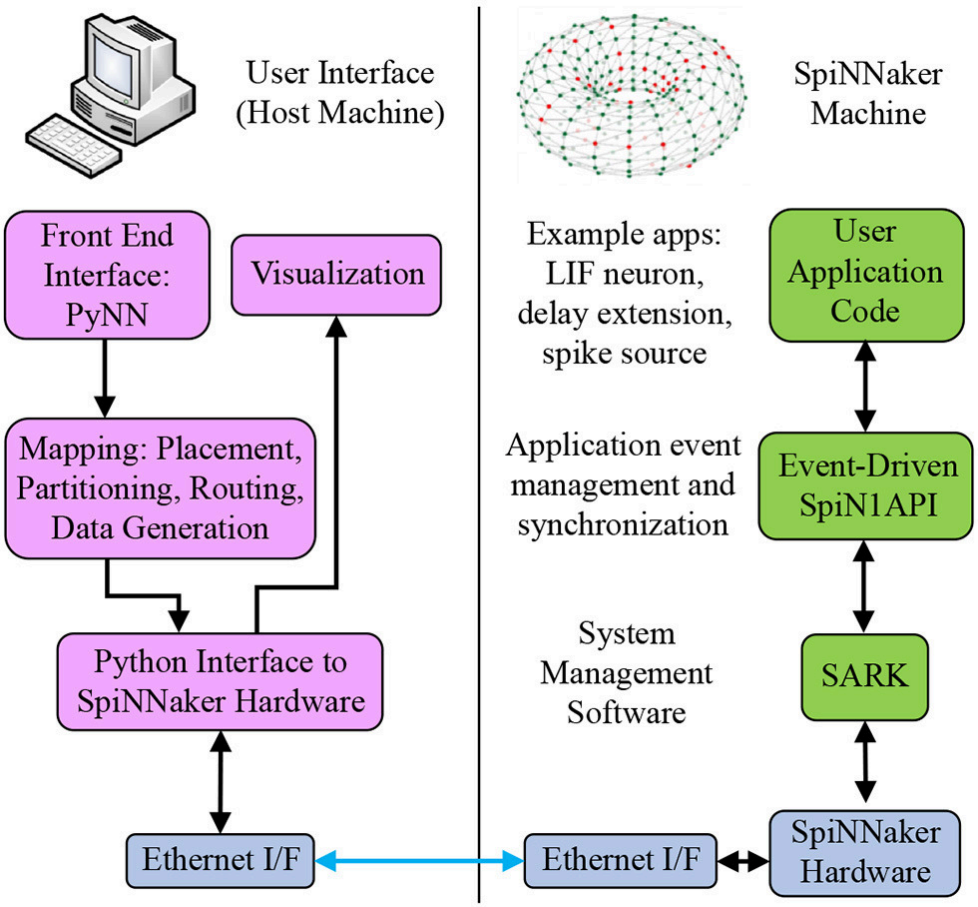
SpiNNaker software stacks. From top left anti-clockwise to top right: users create SNN models on host via the PyNN interface; the sPyNNaker Python software stack then translates the SNN model into a form suitable for a SpiNNaker machine, and loads the appropriate data to SpiNNaker memory via Ethernet; sPyNNaker applications, built on the SARK system management and SpiN1API event-driven processing libraries, use the loaded data to perform realtime simulation of neurons and synapses.

The sPyNNaker API is comprised of two software stacks as shown in Figure 2: one running on host predominantly written in Python, the other running on the SpiNNaker machine written in C. Users create an SNN model via the PyNN interface, which is then preprocessed via the Python sPyNNaker software into a form suitable for a SpiNNaker machine. Simulations execute on the SpiNNaker machine via an event-driven operating system, and on simulation completion results data are extracted back to host for post-processing and visualization via the PyNN API. This section details the software implementation of these stages, beginning with a summary of the SNN-specific aspects of preprocessing (for further explanation of the Python-based preprocessing stages readers are referred to Rowley et al., 2018). Simulation execution on the SpiNNaker machine is then covered in detail, including an overview of the event-driven operating system SpiN1API. The software framework for defining neuron and synapse models is then introduced, followed by an overview of existing implementations. Finally, auxiliary applications are discussed, including generation of input spikes to drive network behavior.

### 3.1. Preprocessing

At the top of the left-hand side stack in Figure 2, users create a PyNN script defining an SNN. The SpiNNaker back-end is specified, which translates the SNN into a form suitable for execution on a SpiNNaker machine. This process includes: mapping of the SNN into an application graph, partitioning into a machine graph, generation of the required routing information, and loading of data and applications to a SpiNNaker machine. Once loading is complete, all core applications are instructed to begin execution, and run for a predefined period. On simulation completion, requested output data are extracted from the machine and made accessible through the PyNN API. Documentation of the complete simulation workflow is beyond the scope of this work, however aspects not detailed here are covered in Rowley et al. (2018) for generic graph-based simulations (extending SpiNNaker beyond neural applications). However, as certain aspects of SNNs have a direct impact on the simulation workflow, a walkthrough of the key points in this process in the context of neural simulation is included below.

**Figure 3:**
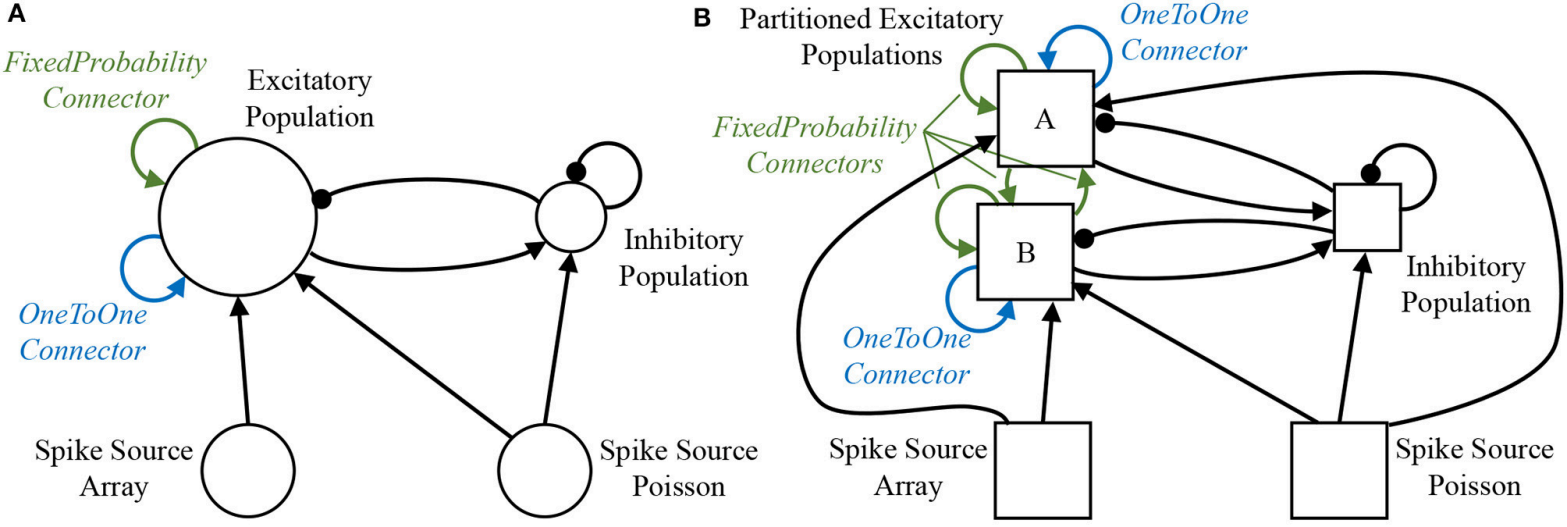
Network partitioning to fit machine resources. **(A)** Application graph generated from interpretation of PyNN script: circles represent PyNN populations, and arrows PyNN projections. **(B)** Machine graph partitioned into vertices and edges to suit machine resources: squares represent populations (or partitioned sub-populations) of neurons which fit on a single SpiNNaker core—hence the model described by the machine graph in **(B)** requires 5 SpiNNaker cores for execution.

A sample SNN is developed as a vehicle by which to describe the stages of preprocessing. A random balanced network is defined according to the PyNN script detailed in Code S1 (Supplementary Material), with the resulting network topology shown in Figure 3A. The network consists of 500 excitatory and 125 inhibitory neurons, which make excitatory and inhibitory projections to one another, respectively. Each population additionally makes recurrent connections to itself with the same effect. Excitatory Poisson-distributed input is included to represent background activity, while predefined spike patterns are injected via a spike source array. The neuronal populations consist of current-based leaky integrate-and-fire (LIF) neurons, with the membrane potential of each neuron in the excitatory population initialized via a uniform distribution bounded by the threshold and resting potentials. The sPyNNaker API first interprets the PyNN defined network to construct an application graph: a vertices and edges view of the neural network, where each edge corresponds to a projection carrying synapses, and each vertex to a population of neurons. This application graph is then partitioned into a machine graph, by subdividing application vertices and edges based on available hardware resources and requirement constraints, ultimately ensuring each resulting machine vertex can be executed on a single SpiNNaker core. From hereon-in the term *vertex* will refer to a machine vertex, and is synonymous with the term sub-population, representing a group of neurons which can be simulated on a single core. An example of this partitioning is shown in Figure 3, where due to its size “Excitatory Population” is split into two sub-partitions (A and B). Figure 3 also shows how additional machine edges are created to preserve network topology between partitions A, B, and the other populations, and how different PyNN connectors are treated differently during this process. For example, a PyNN *OneToOneConnector* connects each neuron in a population to itself. This results in both partitions A and B having a machine edge representing their own connections, but with no edge required to map the connector from one sub-population to the other. Conversely, the PyNN *FixedProbabilityConnector* links neurons in the source and target populations based on connection probability, and hence requires machine edges to carry all possible synaptic connections (e.g., both between vertices A and B, and to themselves).

Once partitioned, the machine graph is placed onto a virtual representation of a SpiNNaker machine to facilitate allocation of chip-based resources such as cores and memory. Known failed cores, chips and board links which compromise the performance of a SpiNNaker machine are removed from this virtual representation, and the machine graph placed accordingly. Chip-specific routing tables are then generated facilitating transmission of spikes according to the machine edges representing the PyNN-defined projections. These tables are subsequently compressed and loaded into router memory (as described in section 2.1). The Python software stack from Figure 2 then generates the core-specific neuron and synapse data structures, and loads them onto the SpiNNaker machine using the SpiNNTools software. Core-specific neuron data is loaded to the appropriate DTCM, while the associated synapse data is loaded into core-specific regions of SDRAM on the same chip, ready for use according to section 3.2. Finally, programs for execution on application cores are loaded to ITCM, with each core executing an initialization function to load appropriate datastructures (from SDRAM) and prepare the core before switching to a *Ready* state. Once all simulation cores are *Ready*, the signal to begin simulation is given to all cores from host, and the SNN will execute according to the processes defined in section 3.2.

### 3.2. SpiNNaker runtime execution

sPyNNaker applications execute SNNs via a hybrid simulation approach, using time-driven neuron updates and event-driven synapse updates, similar to that discussed in Morrison et al. (2005). This neuron update scheme provides a flexible framework in which to embed a range of neuron models, and is of comparable efficiency to event-based approaches when considering biologically representative spike rates. Synapse events are handled efficiently, with no intermediate information required to update synaptic state between presynaptic neuron spikes, which are relatively infrequent on the order of 1Hz in biological networks. Cores executing sPyNNaker applications hold neuron state variables in local DTCM, allowing efficient access to the required datastructures for the periodic time-driven neuron update. Spike transmission between cores is via the Address Event Representation (AER) model (Mead, 1989), with neuronal action potentials communicated as multicast packets, with their key containing only the source neuron ID (in the remainder of this work the terms: action potential, spike, and packet are synonymous). Each packet can be delivered to multiple locations simultaneously via the SpiNNaker routing fabric, replicating the one-to-many connectivity of an axon. Processing of the packet is performed by the core simulating the postsynaptic neuron, which contains functions to evaluate the spike-based synaptic contribution using only the packet key. Due to the potentially large fan-in to a neuron, memory constraints prevent storage of synaptic data in DTCM. Therefore, the source neuron ID is used to locate the associated synaptic data stored in the relatively large but slower SDRAM memory, and copy it locally on spike arrival to facilitate evaluation of the contribution to the synaptic state.

This section focuses on the deployment of this simulation approach within a single core modeling a sub-population of neurons, such as “Excitatory A” in Figure 3B. It includes: an overview of the underlying event-based operating system and low-level software libraries; a discussion of the execution framework and key software functions (also known as callbacks); and finally a discussion of runtime operation giving readers insight into the processing hierarchy. A subsequent discussion of the effect of this simulation approach on neuron and synapse model dynamics is given in section 3.3.

#### 3.2.1. Operating system and low-level libraries

**Figure 4:**
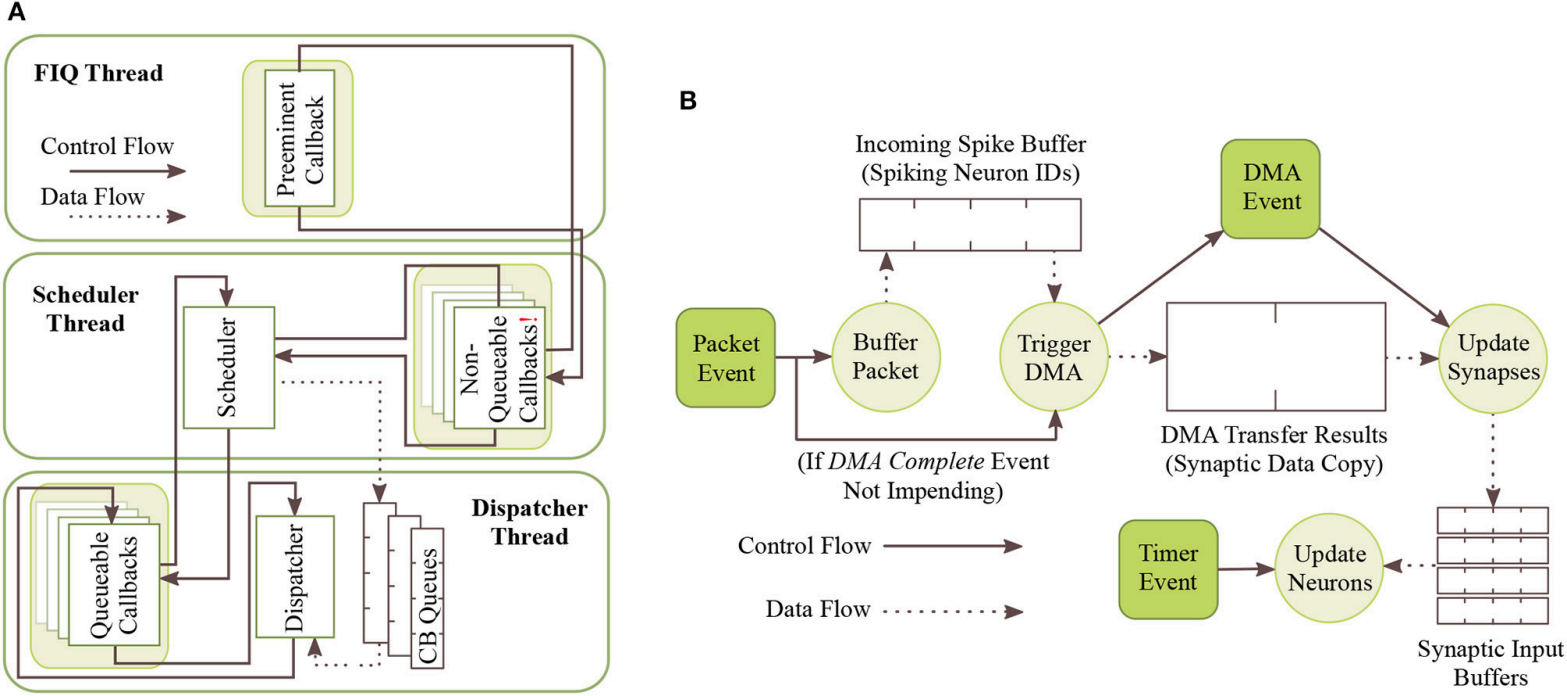
SpiNNaker realtime OS. **(A)** SpiN1API multi-threaded event-based operating system: scheduler thread to queue callbacks; dispatcher thread to execute callbacks; and FIQ thread to service interrupts from high-priority (-1) events. **(B)** Events and associated callbacks for updating neuron state variables, and processing incoming packets representing spikes into synaptic input. Figures reproduced with permission from Sharp et al. (2011, 2012).

To abstract away the complexities of low-level programming and enable focusing on the task of neural modeling, sPyNNaker applications are compiled against the SpiNNaker Application Runtime Kernel (SARK) (Temple, 2016), and the event-driven library SpiN1API (Sharp et al., 2011; SpiNNaker, 2011a). SARK provides low-level hardware management, simplifying interaction with the DMA, Network Interface and Communications controllers. SpiN1API provides an event-based operating system, as shown in Figure 4, with three processing threads per core: one for task scheduling, one for task dispatch, and one to service fast interrupt requests (FIQ). SpiN1API also provides the mechanism to link software callbacks to hardware events, and enables triggering of actions such as sending a packet to another core and initiating a DMA. Callbacks are registered with different *priority* levels ranging from -1 to 2 depending on their desired function, with lower numbers scheduled preferentially. Callback tasks of priority 1 & 2 can be queued (in queues of maximum length 15), with new events added to the back of the queue. Callbacks of priority -1 and 0 are not queued, but instead pre-empt tasks assigned higher priority level numbers. Operation of this system follows the flow detailed in Figure 4A. The scheduler thread places callbacks in queues for priority levels 1 and above, and the dispatcher picks these callbacks and executes them based on priority. When the dispatcher is executing a callback of priority 1 or higher, and a callback of priority 0 is scheduled, this task pre-empts that currently being executed causing it to be suspended until the higher priority callback has completed. Callbacks of priority −1 use the FIQ thread to interact with the scheduler and dispatcher, enabling fast response and pre-emption of priority 0 and above tasks. Pointers are stored allowing fast access to the callback code, and the processor switches to FIQ banked registers to avoid the need for stacking (Sloss et al., 2004), optimizing the response time of priority −1 callbacks. However, this optimized performance limits the application to registering only a single −1 priority event and callback.

In sPyNNaker applications modeling systems of neurons and synapses, callbacks are registered against hardware events: *timer, packet received*, and *DMA complete*; and a software-triggered *user* event, as shown in Table 1. The associated callbacks facilitate the periodic updating of neuron state, and the event-based processing of synapses when packets representing spikes arrive at a core. These events (squares) and their callbacks (circles) are shown schematically in Figure 4B. The function timer_callback evolves the state of neurons in time, and is called periodically against *timer* events throughout a simulation. A *packet received* event triggers a _multicast_packet_received_callback, which reads the packet to extract and transfer the source neuron ID to a spike queue. If no spike processing is currently being performed, the software-triggered *user* event is issued, and in turn executes a user_callback which reads the next ID from the spike queue, locates the associated synaptic information stored in SDRAM, and initiates a DMA to copy it into DTCM for subsequent processing. Finally the _dma_complete_callback is executed on a *DMA complete* event, and initiates processing of the synaptic contribution(s) to the postsynaptic neuron(s). If on completion of this processing there are items remaining in the input spike queue, this callback initiates processing of the next spike: meaning this collection of callbacks can be thought of as a spike processing pipeline.

**Table 1:** Hardware (and single software) events, along with their registered callback and associated priority level.

**Event**	**Callback**	**Priority**	**Pre-empts Priority**
*Packet Received*	_multicast_packet_received_callback	−1	0, 1, 2
*DMA Complete*	_dma_complete_callback	0	1, 2
*Timer*	timer_callback	2	–
*User* (Software)	user_callback	0	1, 2

#### 3.2.2. Time-driven neuron update

**Figure 5:**
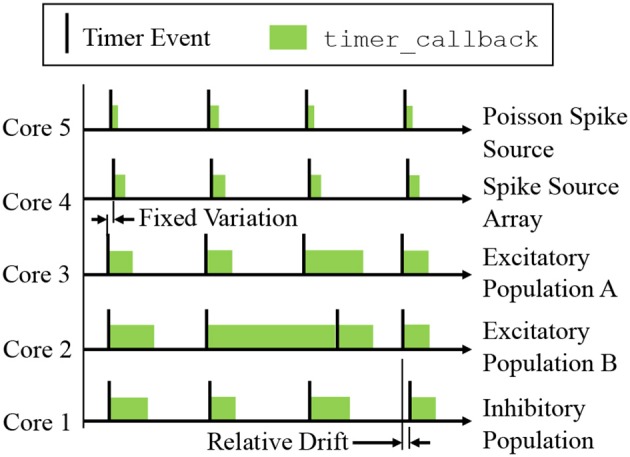
Time-driven updates by neuron cores simulating the network in Figure 3B: periodic *timer* events trigger callbacks advancing neuron states by Δ*t*. Cores can be out of phase due to communication of the start signal, and relative drift can occur due to manufacturing variability between boards. Note that state update times vary with the level of additional spike processing within a simulation timestep, however cores which experience high levels of spike activity delaying the subsequent timer_callback can catch up during subsequent periods of lower spike activity (as shown by Core 2).

A sPyNNaker simulation typically contains multiple cores, each simulating a different population of neurons (see Figure 3B). Each core advances the states of its neurons in time via an explicit update scheme with fixed simulation timestep (Δ*t*). When a neuron is deemed to have fired, packets are delivered to all cores that neuron projects to, and processed in realtime by the postsynaptic core to evaluate the resulting synaptic contribution. Therefore, while all cores operate asynchronously, it is desirable to advance neurons on all cores approximately in parallel in order to march forward a simulation coherently. All cores in a simulation therefore start synchronized, and register *timer* events with common frequency, with the period between events defined by a fixed number of clock cycles, as shown in Figure 5. All cores will therefore initiate a *timer* event and execute a timer_callback to advance the state of their neurons approximately in parallel, although the system is asynchronous as there is no hardware or software mechanism to synchronize cores. Individual update times may vary due to any additional spike processing (see section 3.2.6), however cores which have additional spikes to process between one pair of timer events can catch up during subsequent periods of lower activity. Relative drift between boards is possible due to slight variations in clock speed (from clock crystal manufacturing variability), however this effect is small relative to simulation times (SpiNNaker, 2011b). Small variations placing core updates slightly out of phase can also occur due to the way the “start” signal is communicated, particularly on larger machines, however again this effect is negligible. A consequence of this update scheme is that generated spikes are constrained to the time grid (multiples of the simulation timestep Δ*t*). It also enforces a finite minimum simulation spike transit time between neurons of Δ*t*, as input cannot be guaranteed to arrive in the current timestep before a neuron has been updated. From the hardware perspective, the maximum packet transit time for the million core machine is ≤ 25μs (assuming 200ns per router (SpiNNaker, 2011b), and a maximum path length of 128).

A design goal of the SpiNNaker platform is to achieve realtime simulation of SNNs, where “realtime” is defined as when the time taken to simulate a network matches the amount of time the network has modeled. Therefore, an SNN with a simulation timestep of Δ*t* = 1ms, requires the period of *timer* events to be set at 200, 000clock cycles (where at 200MHz each clock cycle has a period of 5ns—see section 2.1). This causes 1ms of simulation to be executed in 1ms, meaning the solution will keep up with wall-clock time, enabling advantageous performance, and interaction with systems operating on the same clock (such as robots, humans and animals). In practice, realtime execution is not always possible, and therefore users are free to reduce the value of Δ*t* in special cases, and also adjust the number of clock cycles between *timer* events. For example, if a neuron model requires Δ*t* = 0.1ms for accuracy, it is common practice to let the period between *timer* events remain at 200, 000clock cycles, to ensure there is sufficient processing time to update the neurons and process incoming spikes (Sen-Bhattacharya et al., 2018). This enforces a slowdown factor of 10 relative to realtime.

**Figure 6:**
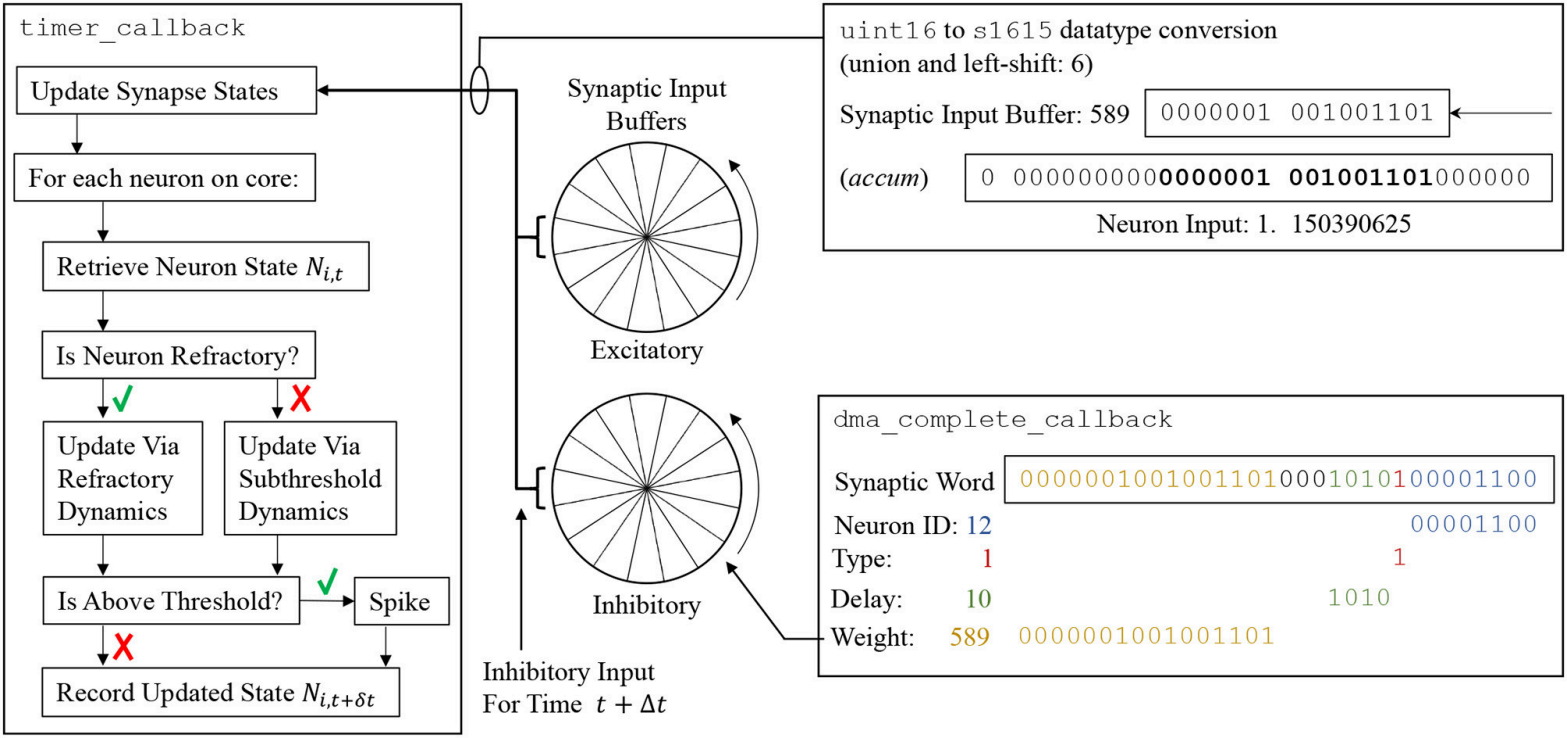
**(Left)** Update flow advancing state of neuron *N*_*i*_ by Δ*t*. **(Center)** Circular synaptic input buffers accumulate scaled input at different locations based on synaptic delay (buffers are rotated one slot at the end of every timestep). **(Right top)** Synaptic input buffer values are converted to fixed-point format and scaled before adding to *N*_*i*_. **(Right bottom)** Decoding of synaptic word into circular synaptic buffer input.

From the perspective of an individual core, each neuron is initialized with user-defined parameters at time *t*_0_ (supplied via a PyNN script). All state variables are then updated one timestep at a time up to the simulation end time *t*_*end*_. The number of required updates and hence *timer* events is calculated based on *t*_*end*_ and the user-defined simulation timestep Δ*t* (which is fixed for the duration of simulation). Each call to timer_callback advances all the neurons on a core by Δ*t* according to Algorithm S1, which is shown schematically on the left-hand side of Figure 6. First the synapse state for all neurons on the core is updated according to the model shaping rule, and any new input this timestep is added from the synaptic input buffers (discussed below). Interrupts are disabled during this update to prevent concurrent access to the buffers from spike processing operations. The states of all neurons on the core are then updated sequentially. An individual neuron state at the current time *N*_*i, t*_ is accessed in memory, and if the neuron is not refractory, its state is updated according to the model characterizing its sub-threshold dynamics (see examples in section 3.3). If it is judged to have emitted a spike, the refractory dynamics are initiated and the router instructed to send a multicast packet to the network. Finally, all requested neuron variables are recorded as belonging to this new timestep (*t*+Δ*t*), and stored in core memory for subsequent extraction by the SpiNNTools software—interrupts are disabled during this process to prevent concurrent access to recording datastructures.

Synaptic input buffers (Figure 6, center) are used to accumulate all synaptic input on a given receptor type, removing the computational cost of managing state variables for individual synapses (as developed in Morrison et al., 2005). Each buffer is constructed from a number of “slots,” where each slot represents input at a future simulation timestep. All input designated to arrive at a particular time is accumulated in the appropriate slot, constraining synapse models to those whose contributions can be summed linearly. A pointer is maintained to the input associated with the proceeding timestep (*t*+Δ*t*). Each neuron update consumes the input addressed by this pointer and then advances it forward one slot (effectively rotating the buffer). When the pointer reaches the last slot it cycles back to the first, meaning these slots continuously represent input over the next *d* timesteps, where *d* is the number of slots. By default the value of *d* is set via a 4-bit unsigned integer, enabling representation of delays up to 16 timesteps (however section 3.4.2 contains information on extending this delay). In the default sPyNNaker implementation a synaptic input buffer is created per neuron, per receptor type, and is a collection of 16 slots each constructed from unsigned 16-bit integers. The use of an integer representation reduces buffer size in DTCM, and also the size of synaptic weights in SDRAM, relative to using standard 32-bit fixed point *accum* type. However, it requires conversion to *accum* type for use in the neuron model calculations—as shown in Figure 6. This conversion is performed via a union and left-shift, the size of which represents a trade-off between headroom and precision. An example shift of 6 is shown, causing the smallest bit of the synaptic input buffer to represent 2^−9^ = 1.953125 × 10^−3^, and the largest 2^7^ = 128, in the *accum* type of the synapse state. Under extreme conditions a buffer slot will saturate from concurrent spike activity, meaning the shift size should be increased. However, the shift is also intrinsic to the weight representation and affects precision, as all weights must be scaled by 2^(15−*shift*)^ before being written as integers to the synaptic matrices discussed in section 3.2.5. For example, in Figure 6 a weight of 1.15nA was converted to 589 on host during generation of synaptic data, but is returned as 1.150390625nA when used during simulation (with a shift of 6). The shift value is currently calculated by the sPyNNaker toolchain to provide a balance between handling large weights, high fan-in and/or presynaptic firing rates, and maintaining precision—see van Albada et al. (2018) for further details.

#### 3.2.3. Receiving a spike

A _multicast_packet_received_callback is triggered by a *packet received* event, raised when a multicast packet arrives at the core. This callback is assigned highest priority (−1), and hence makes use of the FIQ thread and pre-empts all other core processing (see Figure 4A). This callback cannot be queued, therefore to prevent traffic backing up on the network this callback is designed to execute quickly, and simply extracts the source neuron ID (from the 32-bit key) and stores it in an input spike buffer for subsequent processing. Note that by default this buffer is 256 entries long, enabling queuing of 256 spikes simultaneously. The callback then checks for activity in the spike processing pipeline, and registers a *user* event if inactive. Pseudo code for this callback is available in Algorithm S2.

#### 3.2.4. Activation of the spike processing pipeline

**Figure 7:**
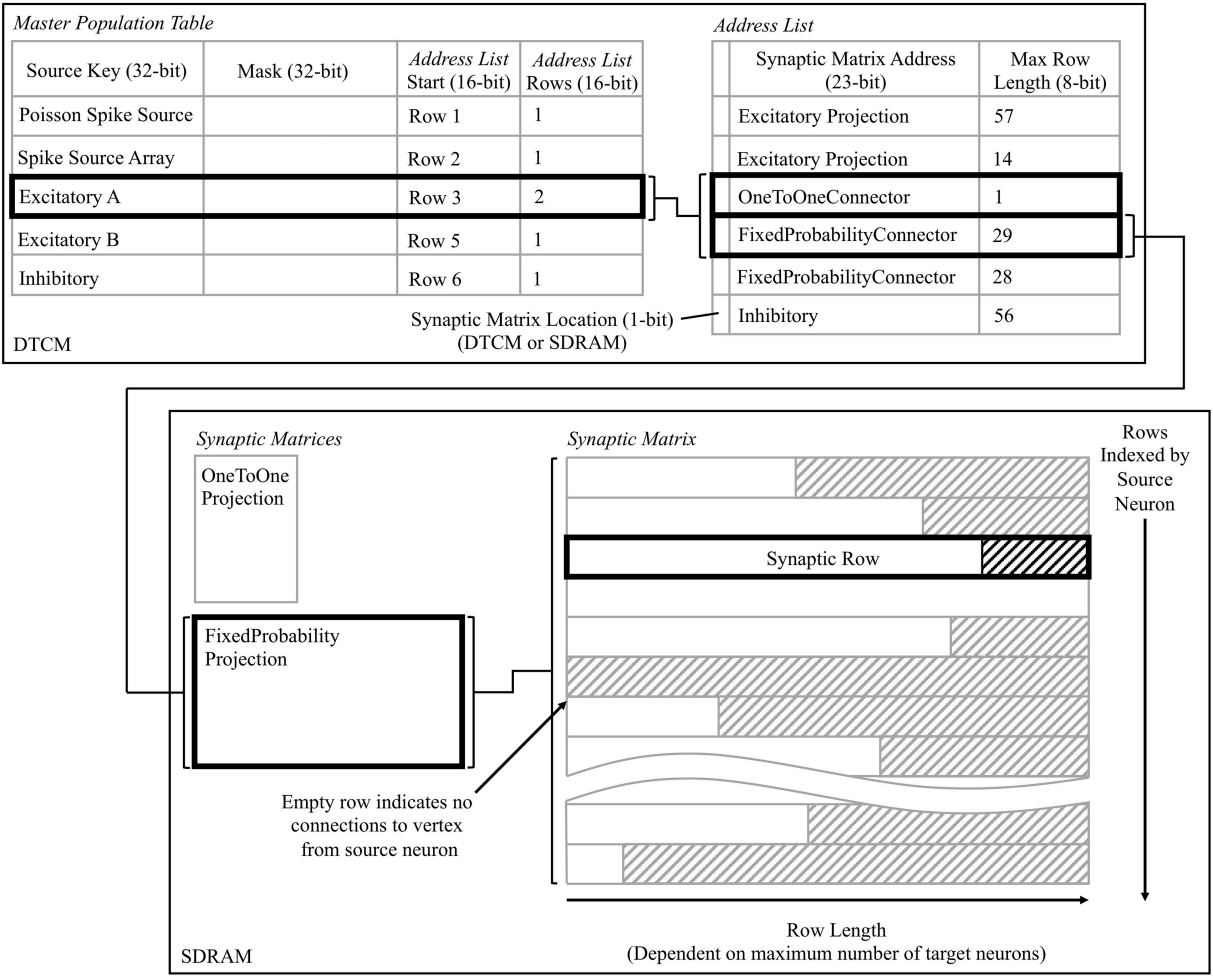
Data structures for processing incoming spikes: *Master Population Table, Address List*, and *Synaptic Matrix*; shown from the perspective of the core simulating the Excitatory A population in Figure 3B. The path in bold represents that taken when a packet is received by Excitatory A, originating from itself, and hence two projections must be processed.

A user_callback callback is triggered by the *user* event registered in a section 3.2.3, and kick-starts the spike processing pipeline. The callback locates in SDRAM the synaptic data associated with the spike ID, and initiates its DMA transfer to DTCM for subsequent processing. Three core-specific data structures are used in this process, the: *master population table, address list*, and *synaptic matrix*. Use of these datastructures is shown schematically in Figure 7, from the perspective of the core simulating the Excitatory A population in Figure 3B, when receiving a spike from the Excitatory A population. The *master population table* is a lightweight list taking a masked source neuron ID as the key by which a source vertex can be identified. Each row pertains to a single source vertex, and consists of: 32-bit key; 32-bit mask; 16-bit start location of the first row in the *address list* pertaining to this source vertex; and a 16-bit value defining the number of rows, where each row in the *address list* represents a PyNN projection. When searching this table the key from the incoming packet is masked using each entry-specific mask before comparing to the entry key. This masks off the individual neuron ID bits, and enables source vertices to simulate different numbers of neurons. The entry keys are masked on host before loading for efficiency, and are structured to prevent overlap after masking and facilitate binary searching. The structure of an *address list* row consists of: a single header bit detailing whether the synaptic matrix associated with this projection is located in DTCM or SDRAM; 32-bit memory address indicating the first row of the synaptic matrix; and an 8-bit value detailing the synaptic matrix row length (i.e., the maximum number of postsynaptic neurons connected to by a presynaptic neuron in a particular projection). Note that synaptic matrix rows are indexed by source neuron ID, and that all rows are padded to the maximum row length to facilitate retrieval, including empty rows for presynaptic neurons not connected to neurons on this core. The row data structure is covered in detail in section 3.2.5.

**Figure 9:**
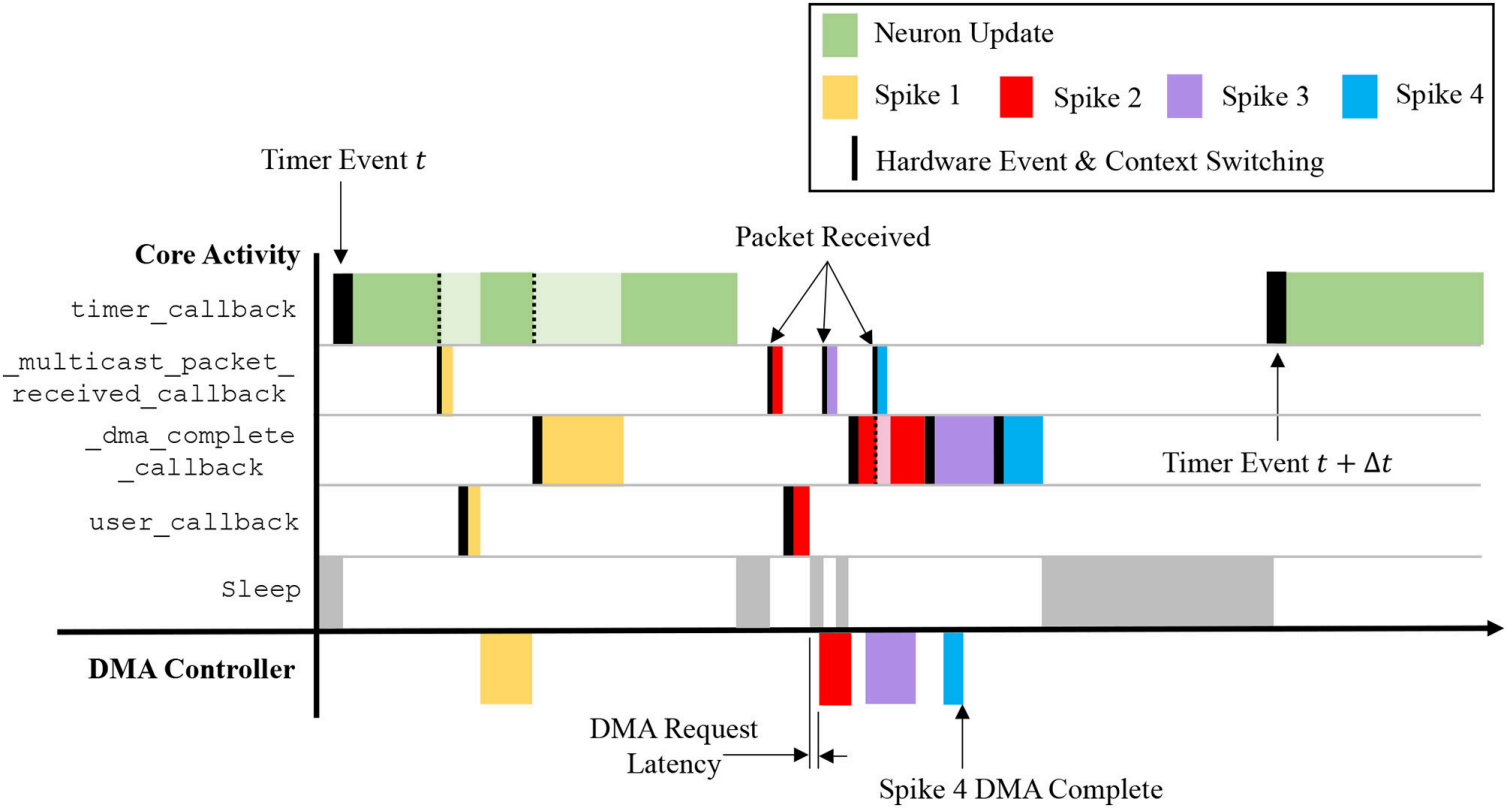
Interaction of callbacks shown over the time period between two *timer* events. Four spike events are processed representing the scenarios: receiving a packet while processing a *timer* event; receiving a packet while the core is idling; and receiving a packet while the spike processing pipeline is active. Note that a lighter color shade indicates suspension of a callback, which is resumed on completion of higher priority tasks.

This callback therefore takes from the input spike buffer the next spike ID to process, and uses it in a binary search of the *master population table* to locate the *address list* regions capturing the projections carrying the spike to this vertex. The SDRAM location and size specified by each row are then used in sequential processing of the projections. For the case shown in Figure 7, searching the *master population table* yields two rows in the *address list*, which in turn define the location of the corresponding synaptic matrices in SDRAM. Each synaptic matrix is indexed according to presynaptic neuron ID, enabling location of the appropriate row to copy to core DTCM for processing of each spike. Details of this row are then passed to the DMA controller to begin the data transfer, marking the end of the callback. This allows the core to return to processing other callbacks, hiding the DMA transfer as shown for “Spike 1” in Figure 9.

#### 3.2.5. Synapse processing

**Figure 8:**
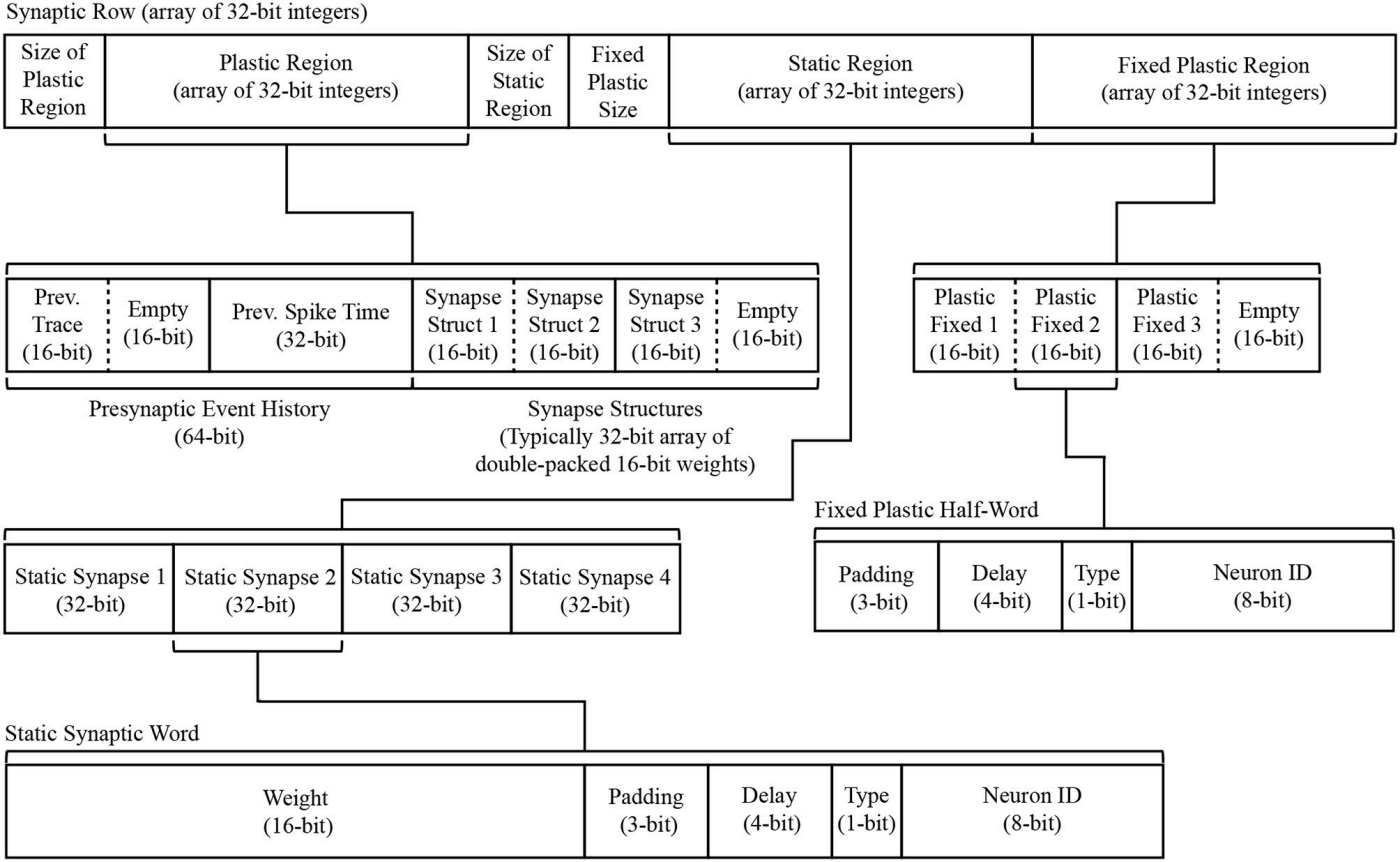
Synaptic row structure with breakdown of substructures for both static and plastic synapses.

On completion of the DMA in section 3.2.4, a *DMA complete* event triggers a _dma_complete_callback, initiating processing of the synaptic row. As described previously, each row pertains to synapses made, within a single PyNN projection, between a single presynaptic neuron and multiple postsynaptic neurons. At the highest level, a synaptic row is an array of synaptic words, where each word is defined as a 32-bit unsigned integer. The row is split into three designated regions to enable identification of static and plastic synapses (connections capable of changing their weight at runtime). The row regions contain: dynamic plastic data; constant fixed plastic data; and static data. Three header fields are also included, detailing the size of each region and enabling easy navigation of the row. A schematic breakdown of the synaptic row structure is detailed in Figure 8. Note that because a PyNN projection cannot be both static and plastic simultaneously, a single row contains only either static or plastic data. Plastic data is intentionally segregated into dynamic and fixed regions to facilitate processing. While all plastic data must be copied locally to evaluate synaptic contributions to a neuron, only the dynamic region—i.e., that changing at runtime—requires updating for use when processing subsequent spikes. Keeping this dynamic data in a separate block facilitates writing back to the synaptic matrix with a single DMA, and writing back less data helps compensate for reduced DMA write bandwidth (relative to read—see section 2.1).

The static region occupies the lower portion of the synaptic row, and is itself an array of synaptic words, where each word corresponds to a synaptic connection between the row's presynaptic neuron and a single postsynaptic neuron. As shown in Figure 8, each 32-bit data structure is split such that the top 16 bits represent the weight, while the lower 16 bits typically split: bottom 8 bits specifying the postsynaptic neuron ID; 1 bit to specify the synapse type (excitatory 0, or inhibitory 1); 4 bits to specify synaptic delay; leaving 3 bits of padding (useful for model customization, e.g., adding additional receptor types). Data defining plastic synapses is divided across the dynamic and fixed regions. Fixed plastic data is defined by a 16-bit unsigned integer, and matches the structure of the lower half of a static synapse (see lower half of Figure 8). These 16-bit synaptic half-words enable double-packing inside the 32-bit array of the synaptic row, meaning an empty half-slot will be apparent if the row targets an odd number of postsynaptic neurons. The dynamic plastic region contains a header defining the *Presynaptic Event History*, followed by a series of *Synapse Structures* capturing the weight of each synapse. Note that for typical plasticity models this defaults to the same 16-bit weight describing static synapses, however *Synapse Structure* can be extended to include additional parameters (in multiples of 16 bits) if required by a given plasticity rule.

A task of the _dma_complete_callback is therefore to convert the synaptic row into individual postsynaptic neuron input. The callback processes the row headers to ascertain whether it contains static or plastic data, adjusts synapses according to a given plasticity rule, and then loops over each synaptic word and extracts its neuronal contribution—pseudo code for this callback is detailed in Algorithm S4. An example of this process for a single static synaptic word is shown in the lower-right of Figure 6, where a synaptic word of [00000010010011010001010100001100] leads to a contribution of 589 to slot 10 of the inhibitory synaptic input buffer for neuron *N*_12_.

#### 3.2.6. Callback interaction

The callbacks described above define how a sPyNNaker application responds to hardware events and updates an SNN simulation. The interaction of these events is a complex process, with the potential to impact the ability of a SpiNNaker machine to perform realtime execution. Figure 9 covers the time between two *timer* events, and shows interaction of spike processing and neuron update callbacks for four scenarios detailed by the arrival of spikes 1–4. The first *timer* event initiates processing of the neuron update, however after completion of approximately one third of the update, the core receives Spike 1, interrupting the timer_callback and triggering execution of a _multicast_packet_received_callback, which in turn raises a *user* event, initiating DMA transfer of the appropriate synaptic information. On completion of the callback, the core returns to the timer_callback, with the DMA transfer occurring in parallel. On completion of the DMA, a _dma_complete_callback is initiated, which processes the transferred synaptic information into neuronal input. The core then returns to the timer_callback, which continues to completion. The core is idle when it receives Spike 2, therefore processing of the spike begins immediately, and the subsequent *user* event and hence DMA request is initiated. While waiting for the data to transfer, Spike 3 is received, and the associated _multicast_packet_received_callback processed. This time, due to the active spike processing pipeline, no *user* event is raised, and instead the DMA for Spike 3 is initiated at the beginning of the _dma_complete_callback triggered by Spike 2. Whilst processing this callback, Spike 4 is received, and the associated _multicast_packet_received_callback interrupts the core to place the packet key in the input spike buffer. This buffer entry is eventually processed at the beginning of the _dma_complete_callback for Spike 3, demonstrating the spike processing pipeline in action. This also shows the benefit of having two hardware “threads” working in parallel, as the core is utilized completely, and the DMA transfer hidden behind the _dma_complete_callback, when the pipeline is active. Finally, after an idle period (where the processor is put to sleep in a low energy state) the next *timer* event is issued at time *t*+Δ*t*.

From Figure 9 it is seen that core processing is dependent on SNN activity. When targeting realtime execution (section 3.2.2), it is important to consider extreme circumstances and how they will affect both the core and global simulation. For example, it is clear from Figure 9 that when a core receives spikes, it can delay completion of the timer_callback due to the assigned callback priorities (as shown in Figure 5). This is a design choice, as it helps maximize core utilization by hiding DMA transfers behind the timer_callback when the spike processing pipeline is inactive. However, in the extreme case spike processing will delay completion of the callback beyond the issuing of the next *timer* event. While the core can potentially catch up this lost time, this scenario has the potential to delay the neuron update beyond a single *timer* event, and ultimately cause any spike packets emitted from this core to be received and processed at the wrong time by the rest of the network. To guard against this, sPyNNaker applications report any occurrences of an overrun, where a timer_callback is not complete before the next *timer* event is raised; and also the maximum number of *timer* events that a single timer_callback overruns. Similar metrics are also reported when the input spike buffer overflows (exceeds 256 entries), and when the synaptic input buffers saturate. Together these metrics provide a window into the ability of a core to handle the required processing within a simulation.

Another important performance consideration when responding to spike packets using prioritized events is the time taken to switch between the associated callbacks. Events are displayed in Figure 9 by solid black lines, the width of which represents the time taken to switch context and begin execution of the callback. The timer_callback takes longest to respond due to queuing of events with priority >0; while the _multicast_packet_received_callback is quickest due to its priority of −1 and use of the FIQ thread. Other chip-level factors can also influence execution, such as SDRAM contention with applications running on adjacent cores. As DMAs are processed in serial bursts, if multiple simultaneous requests are received by the SDRAM controller, there may be latency in beginning the DMA for some cores, and a reduced rate of transfer (see section S1.2 in Supplementary Material for further information).

### 3.3. Neural modeling

At the heart of a sPyNNaker application is the solution of a series of mathematical models governing neural dynamics. It is these models which determine how incoming spikes affect a neuron and when a neuron itself reaches threshold. While the preceding section described the underlying event-based operating system facilitating simulation and interaction of neurons, this section focuses on the solution of equations governing neural state, and how they are structured in software.

#### 3.3.1. Software structure

PyNN defines a number of standard cell models, such as the leaky integrate and fire (LIF) neuron, and the Izhikevich neuron. Implementations of these standard models are included in sPyNNaker, however the API is also designed to support users wishing to extend this core functionality and implement neuron models of their own. To facilitate this extension the model framework is defined in an object-oriented fashion, through the use of C code on the SpiNNaker machine. This modular approach provides structure and aids code re-use between different models (e.g., sharing of a synaptic plasticity rule between different neuron models). A neuron model is built from components:

– *synapse_type*, defining how synapse state evolves between presynaptic spikes, and how contributions from new spikes are added to the model. A fundamental requirement is that multiple synaptic inputs can be summed and shaped linearly, such as the α-kernel (Destexhe et al., 2014).
– *neuron_model*, implementing the sub-threshold update scheme and refractory dynamics.
– *input_type*, governing the process of converting synaptic input into neuron input current. Examples include current-based and conductance-based formulations (Dayan and Abbott, 2005).
– *threshold_type*, defining a system against which a neuron membrane potential is compared to adjudge whether the neuron has emitted a spike.
– *additional_input_type*, offering a flexible framework to model intrinsic currents dependent on the instantaneous membrane potential, and potentially responding discontinuously on neuron firing (such as the Ca^2+^-activated K^+^ current described in Liu and Wang, 2001).

The individual model components each produce a subset of the neuron and synapse dynamics, and are therefore the entry point for a user looking to deploy a custom neuron model^3^. In-keeping with the aforementioned software stacks in Figure 2, interfaces to each component are written in both Python and C. A single instance of each component is collected via a C header file, and compiled against the underlying operating system described in section 3.2 to generate a runtime application. Python classes for each component facilitate user-interaction with each part of the model, enabling setting of parameter values and initial conditions from a PyNN SNN script.

The runtime execution framework calls each component as part of the timer_callback, as detailed in Algorithm S1 (Supplementary Material) and shown schematically in Figure 6. First the synaptic state is advanced forward in time by a single simulation timestep, using the functions defined by the *synapse_type* component. Core interrupts are disabled during this process to prevent concurrent access of the synaptic input buffers from a _dma_complete_callback. Interrupts are re-enabled when all synaptic states for all receptor types for all neurons on a core have been updated. Each neuron then has its state advanced by Δ*t*. The *input_type* component is called first, converting the updated synaptic state into neuron input current. This includes separate excitatory and inhibitory components, with core implementations capable of handling both current- and conductance-based formulations. The *additional_input* component is then evaluated to calculate the level of any intrinsic currents. The synaptic and intrinsic currents, together with any background current, are then supplied to the *neuron_model* component which subsequently marches forward the neuron state by Δ*t*. The neuron membrane potential is now passed to the *threshold_type* component which tests whether the neuron has fired. If the neuron is above threshold a number of actions are performed: a refractory counter begins to instigate any refractory period; the *additional_input* is notified of the spike to allow updating of appropriate state variables; and finally the core is instructed to send a multicast packet to the router with the neuron ID as key.

#### 3.3.2. Leaky integrate and fire neuron

The sPyNNaker implementation of a current-based leaky integrate and fire neuron (LIF) is described by the hybrid system in Equations (1) and (2). The sub-threshold dynamics are governed according to Equation (1), where *V* is the membrane potential, *I* the input current (combining synaptic, intrinsic and background input), *R*_*m*_ the membrane resistance, τ_*m*_ the membrane leak time constant, and *E*_*l*_ the membrane leak (resting) potential.

(1) $$\begin{array}{ll} \frac{dV}{dt} & =-\frac{V-\left(E_{l}+R_{m}I\left(t\right)\right)}{\tau_{m}}\;\text{if}V>V_{\theta },V=V_{reset} \end{array}$$

(2) $$\begin{array}{ll} \frac{dI_{syn}}{dt} & =-\frac{I_{syn}}{\tau_{syn}}+\delta \left(t-t^{j}\right) \end{array}$$

If *V* exceeds the threshold level *V*_θ_, the neuron is reported to have spiked and *V* is set to the reset potential *V*_*reset*_ for the refractory period duration *t*_*r*_. Synaptic currents *I*_*syn*_ are modeled according to Equation (2), where τ_*syn*_ is the synaptic time constant (independent value for each receptor type), and the delta function represents addition of a step change in input from the weight of an incoming spike.

The sPyNNaker implementation embeds Equation (2) in a *synapse type* component, providing mechanisms to update the input current both between spikes (i.e., when the synaptic input buffer contribution is zero), and on spike arrival. Exact integration is used to update the synapse state during the periodic neuron update, with step changes made from synaptic input buffer contributions according to Equation (3).

(3) $$\begin{array}{l} I_{t+1}=I_{t}e^{-\frac{\Delta t}{\tau_{syn}}}+\Sigma_{j}w_{ij}\delta \left(t-t_{j}\right) \end{array}$$

The constant factor $e^{-\frac{\Delta t}{\tau_{syn}}}$ is pre-calculated before loading to the SpiNNaker machine to avoid evaluation at runtime, as both the divide and exponential operations are relatively expensive on the ARM968 (≈100 clock cycles each). A *neuron model* component captures the neuron state update mechanism, which solves Equation (1) via exponential integration (Rotter and Diesmann, 1999) and assuming the change in current over the timestep is small (Dayan and Abbott, 2005), yielding the update function in Equation (4).

(4) $$\begin{array}{l} V_{t+1}=E_{l}+R_{m}I_{t+\Delta t}-e^{-\frac{\Delta t}{\tau_{m}}}\left(E_{l}+R_{m}I_{t+\Delta t}-V_{t}\right) \end{array}$$

To compensate for this assumption, *w*_*ij*_ is decayed before adding to the synapse to ensure the total charge input to a neuron matches the exact solution (van Albada et al., 2018). Static thresholding defined via the *threshold type*, compares the instantaneous membrane potential to the threshold level *V*_θ_.

#### 3.3.3. Izhikevich neuron

The Izhikevich neuron model (Izhikevich, 2003), allows reproduction of biologically observed neuronal characteristics such as spiking and bursting. Its dynamics follow a type of “quadratic integrate and fire” model, as detailed in Equation (5)

(5) $$\begin{array}{ll} \frac{dv}{dt} & =0.04v^{2}+5v+140-u+I\left(t\right)\\ \frac{du}{dt} & =a\left(bv-u\right) \end{array}$$

(6) $$\text{if}v\geq V_{\theta },\text{then}\{\begin{array}{l} v\leftarrow c\\ u\leftarrow u+d \end{array}$$

where *v* and *u* are dimensionless variables representing the membrane potential and a recovery variable, respectively. Dimensionless parameters *a*, *b*, *c*, and *d* are used to tune the model dynamics, and *I* represents combined background, intrinsic and synaptic currents. If *v* exceeds a threshold *V*_θ_, *v* and *u* are reset according to Equation (6).

The sPyNNaker implementation of this model uses the same *synapse type*, current-based *input type*, and static *threshold type* components as the aforementioned LIF implementation. However, updating the neuron state and hence solving the system defined by Equation (5) requires numerical integration. A range of solvers were explored with fixed-point datatypes in Hopkins and Furber (2015), with the RK-2 midpoint preferred as a trade-off between speed and accuracy. The resulting explicit update scheme is detailed in Equation (7).

(7) $$\begin{array}{ll} \theta & =140+I_{t+\Delta t}-u_{t}\;\alpha =\theta +(5+0.04v_{t})v_{t}\\ \eta & =\frac{h}{2}+v_{t}\;\beta =-\frac{h}{2}a(u_{t}+b(v_{t})\\ v_{t+\Delta t} & =v_{t}+h(\beta +(0.04\eta +5)\eta +\theta \\ u_{t+\Delta t} & =u_{t}+ah(b\eta +\beta -u_{t}) \end{array}$$

While it is hard to recognize the original equations in this form, refactoring of the update scheme and algebraic manipulation leads to several improvements in the implementation. The use of intermediate variables not only enables compiler optimizations improving speed and code size, but also helps prevent over/underflow of the *accum* datatype during intermediate calculations (Hopkins and Furber, 2015).

### 3.4. Auxiliary application code

While neuron-simulating applications capture the core operations of an SNN, several additional sPyNNaker applications are required to generate network input and facilitate network operation. These single-core applications are built following similar principals to those defined in section 3.2, responding via the same event-based operating system to send and receive packets, and interact with neuron cores. They are embedded in the machine graph during network preprocessing, and loaded onto a SpiNNaker machine together with configuration data.

#### 3.4.1. Spike input generation

Generating spikes is an integral part of SNN simulation. It enables modeling of network response to specific patterns of spikes, and input representing adjacent brain regions or background noise. The sPyNNaker API includes two applications for spike generation: *Spike Source Array*, and *Poisson Spike Source*. These applications are built from compiled C and require a single SpiNNaker core per instance. They follow *timer* events in parallel (but asynchronously) with neuron-simulating cores, and send multicast packets representing spikes as discussed previously. These applications do not receive spikes, and hence have their functionality encoded entirely in callbacks registered against *timer* events. As with all sPyNNaker applications, a corresponding Python class enables construction of a spike generator in a PyNN script, and allows configuration data to be specified and subsequently loaded to a SpiNNaker machine.

The *Spike Source Array* application contains a population of neuron-like units which emit spikes at specific times (see Code S1). The times and keys to emit are stored in SDRAM and only copied into local DTCM when required during execution. The buffer of times/keys is pre-loaded up to memory limits and can be replenished during execution by sending requests to the host, although this is limited by the bandwidth of the on-board Ethernet. Callbacks issued on *timer* events (corresponding to timestep updates on neuron cores) then send packets to the router at the prescribed times.

The *Poisson Spike Source* application emits packets according to a Poisson distribution about a given frequency. A population of neuron-like units is specified, each of which can be assigned an individual mean firing rate (see Code S1). At runtime, periodic *timer* events trigger a callback every simulation timestep Δ*t*, which assesses whether the core should send a packet to the router representing a spike. A distinction is made between slow and fast Poisson spike sources based on whether they emit fewer or greater than 1 spike per Δ*t*. For fast spike sources, the number of spikes to send between *timer* events is calculated, and the corresponding packets sent interspersed with random delays. This random spacing reduces the chance of synchronized spike arrival at postsynaptic cores, easing pressure on both the source and target routers. For slow sources, after each spike, an inter-spike interval is evaluated in multiples of Δ*t*, which is then counted down between sending packets. For fast spike sources, the postsynaptic core is likely to retrieve from SDRAM the same pieces of synaptic matrix many times during a simulation. Therefore, to remove the overhead of the DMA, a mechanism is included to store the synaptic matrices from fast spike sources in DTCM.

#### 3.4.2. Simulating extended synaptic delays

While there is a mechanism in the synaptic row to account for delays of up to 16Δ*t*, it can be necessary to prescribe longer delays (particularly when Δ*t* is small). To account for this case, an application called a *delay extension* is created (van Albada et al., 2018), running on an adjacent core. Packets representing spikes exhibiting a delay ≥16Δ*t* are routed to the core running this application, which subsequently sends new spikes targeting the postsynaptic core after a sufficient portion of the delay has elapsed such that any remaining delay can be handled within the synaptic row.

Two datastructures are used to manage delay handling: a “delay stage configuration” is generated during preprocessing, and captures the size of delay associated with each presynaptic neuron; and a “spike counter” registers the time and presynaptic neuron of incoming spikes. Two callbacks are used in the *delay extension*, registered against *packet received* and *timer* hardware events. On packet arrival the first callback extracts the presynaptic neuron ID to an input spike buffer, similar to the process described in section 3.2.3. The second callback is executed on *timer* events occurring in parallel (but asynchronously) with those on neuron processing cores. The callback processes any spikes received since the previous *timer* event, taking entries from the input spike buffer and using them to update the spike counting datastructure to register the incoming spikes against multiples of the number of synaptic input buffer slots on the corresponding postsynaptic core. There are typically 16 such slots, where in this context a collection of 16 slots is referred to as a “delay stage.” A second data structure captures how many delay stages each spike should be held for before being released to the postsynaptic core. Therefore, using these two data structures it is possible to assess the incoming spikes to calculate the corresponding outgoing spike times, and hence schedule the necessary spikes for distribution to the network.

While this application solves the problem of simulating extended delays, it cannot do so indefinitely and an effective new upper limit of 144Δ*t* is enforced due to DTCM constraints. It should also be noted that this mechanism introduces additional overhead to the system: an extra core is required to run the application; and two packets are now required to transmit a spike. The postsynaptic core also performs additional processing during look-up of the source vertex in the *master population table*. An additional row must be included to identify spikes traveling directly from the presynaptic core, and also those sent from each individual delay stage of the *delay extension*. This increased *master population table* size can be costly to search, and detrimental for realtime performance (see section 4.2).

## 4. Results

This section demonstrates results from simulations executed through the sPyNNaker API. The random balanced network used as a demonstration vehicle throughout this work is presented first, demonstrating realtime operation of small scale networks. The section then concludes with results from profiling operations detailed in section 3.2, in order to characterize system performance.

### 4.1. Simulation of a random balanced network

**Figure 10:**
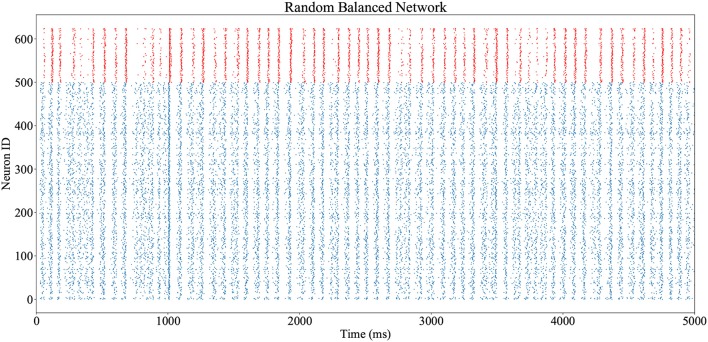
Output spike trains from realtime execution of the random balanced network of Figure 3A, from the PyNN script of Code S1. Spikes from inhibitory neurons are shown in red, and excitatory neurons in blue. The excitatory population receives an additional stimulus at *t* = 1s.

The network shown in Figure 3 and described in Code S1 (Supplementary Material) is executed on a SpiNNaker machine, utilizing 5 cores. Execution is realtime, with 5 s of simulation performed over 5 s of wall-clock time, and all spikes delivered and processed successfully. Output spike trains are accessed via the PyNN interface, and plotted in Figure 10 (excitatory neuron spikes in blue, inhibitory in red). Network oscillations of ≈15Hz are observed throughout, with additional stimulation from the prescribed spike source input at *t* = 1s.

### 4.2. Performance profiling

**Figure 11:**
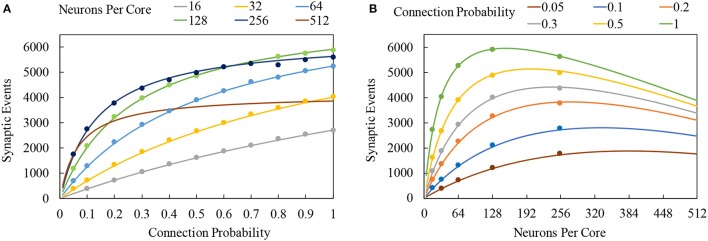
Total number of synaptic events which can be processed between *timer* events: solid lines represent cost model predictions, while markers show measurements taken from sPyNNaker simulations. **(A)** Variation of total synaptic events with connection probability for a range of simulated neurons per core. **(B)** Variation of total synaptic events with neurons per core for a range of connection probabilities.

Realtime performance of a sPyNNaker application is principally governed by: the number of neurons simulated on a core; the complexity of the underlying neuron and synapse models; the peak incoming spike rate to a core during simulation; and the number of neurons each spike targets. To understand how these factors interact, a comprehensive profiling analysis is performed in section S1 (Supplementary Material). This led to development of the cost model in Equation (8), predicting the total number of synaptic events a core can process between *timer* events while maintaining realtime execution. This model assumes a continuously-active pipeline (which is shown to be valid by the results in Figure 11), and that all spikes targetting the core will project to the same number of neurons. Subscript *n* refers to neuron processing contributions; while *s* refers to spike contributions, with *f*, *s*, and *l* referring respectively to the first, subsequent and last spikes in an active pipeline.

(8) $$\begin{array}{l} E_{s}=nP\left(\frac{t_{p}-\left(m_{n}n+c_{n}\right)-\left(m_{s,f}nP+c_{s,f}\right)-\left(m_{s,l}nP+c_{s,l}\right)}{m_{s,s}nP+c_{s,s}}+2\right) \end{array}$$

The time taken to update the state of neurons (*m*_*n*_*n*+*c*_*n*_) is subtracted from the period between *timer events* (*t*_*p*_) to give the time available for processing spikes. The costs of processing the first and last spikes in the pipeline are then subtracted from this time (compensated for by the 2), and the remainder is divided by the cost of processing an intermediate spike (*m*_*s, s*_*nP*+*c*_*s, s*_), to give the total number of spikes which can be processed. This total number of spikes is then multiplied by the number of neurons *n* and connection probability *P*, to give the total synaptic events *E*_*s*_ (as discussed in section 2.1).

Model coefficients for current-based LIF and Izhikevich neurons are developed in section S1, and given in Table 2. The difference of *m*_*n*_ for the two neurons models highlights the additional operations introduced by Equation (5), relative to Equation (1). However, this demonstrates that with careful consideration, SpiNNaker is able to update neurons with dynamics beyond those of a LIF neuron, despite the constraint of fixed-point arithmetic, and with only limited software libraries and processor speed.

**Table 2:** Cost model parameters for LIF and Izhikevich neuron models and static synapse processing.

**Paramter**	**Value**
*m*_*n*_ (LIF)	1.015μs / neuron
*c*_*n*_ (LIF)	3.235μs
*m*_*n*_ (IZK)	1.450μs / neuron
*c*_*n*_ (IZK)	3.231μs
*m*_*s, f*_	0.126μs / synaptic word
*c*_*s, f*_	6.567μs
*m*_*s, s*_	0.115μs / synaptic word
*c*_*s, s*_	3.96μs
*m*_*s, l*_	0.115μs / synaptic word
*m*_*s, l*_	2.48μs

Using the parameters of Table 2, it is possible to estimate the interaction of neuron and spike processing for a range of SNN conditions. Constructing an SNN with single source neuron population, targetting a neuron population of variable size (16–512), with a connecting projection of variable probability (0.05–1.0), it is possible to evaluate the total number of synaptic events which can be processed while maintaining realtime execution. Predictions are generated for LIF neurons, assuming a single activation of the spike processing pipeline, and that all spikes target the same number of neurons. Model predictions are verified against sPyNNaker simulations containing SNNs replicating the network and assumptions above. Simulations are executed where the spike source emits increasing numbers of spikes to the postsynaptic neuron population within each timestep, in order to find the limiting case beyond which realtime execution can no longer be honored. Test data is plotted as circular markers in Figure 11, together with model predictions shown by continuous lines. Figure 11A shows the total number of synaptic events against connection probability for a range of numbers of neurons simulated on a single core. For all cases, increasing connection probability increases the total synaptic events, as each spike will now target greater numbers of neurons. As the number of neurons on a core increases, this behavior is reinforced up to maximum of 5,922 synaptic events when each spike targets 128 neurons with a connection probability of 1. However Figure 11B, which displays the same data plotted against numbers of neuron per core for a range of connection probabilities, shows this is a limiting case. Increasing beyond 128 neurons per core for full connectivity shows a reduction in the total synaptic events, as the additional time required to process neuron state updates leaves less time available to process incoming spikes. At lower connectivity, this performance peak occurs at higher numbers of neurons: e.g., at 20% connectivity the maximum number of synaptic events is achieved for ≈255 neurons per core. Also included in Figure 11 are performance predictions for a core simulating 512 neurons. Although sPyNNaker does not currently support simulating this many neurons on a single core, it is useful to use Equation (8) to predict the effect of further increasing the number of neurons. It is seen that for the model coefficients in Table 2, performance is comparable to simulating 255 neurons per core at low connection probabilities, however total synaptic events plateaus around 30% connectivity (Figure 11A), processing only 68% of the synaptic events achievable when running 255 neurons per core with a fully-connected network.

Inspection of Equation (8) shows several coefficients govern overall performance. For example, in all cases the variable component of updating a single neuron dominates the associated fixed processing cost, meaning *m*_*n*_ plays the governing role in determining the remaining time available for processing incoming spikes. In terms of how many spikes can be processed, it is the subsequent spikes within an active pipeline which dominate throughput (as the first and last spikes are assumed to occur only once). At high connection probabilities and neurons per core *m*_*s, s*_*nP*>*c*_*s, s*_, meaning the variable per-target-neuron cost (*m*_*s, s*_) dominates the total number of spikes which can be processed. Conversely, when connection probabilities and neurons per core are low, the fixed cost of processing a spike within the pipeline (*c*_*s, s*_) dominates.

The components of these coefficients have been profiled in detail for a range of neuron models and network topologies in section S1 (Supplementary Material). The coefficient *m*_*n*_ is a function of neuron model and output spike rates, while *m*_*s, s*_ is dependent on processing a synaptic row into synaptic input buffer contributions. The coefficient *c*_*s, s*_ is predominantly dependent on the spike processing pipeline, with major contributions coming from: locating the memory address of synaptic information associated with an incoming spike; initializing a DMA of this data; and responding to interrupts and context switching within the pipeline. From Equation (8) it is seen that reducing *m*_*n*_ has the effect of enabling higher numbers of neurons per core, unlocking additional performance from low connectivities through targetting more neurons for the same probability of connection. Reducing *m*_*s, s*_ has the effect of increasing the number of spikes which can be processed, shifting the peak in maximum synaptic events of Figure 11B to higher values of neurons per core for the same probability of connection. Reducing *c*_*s, s*_ improves performance across all connection probabilities, but shifts the peak of maximum synaptic events in Figure 11B to lower numbers of neurons per core—i.e., improving performance for sparsely-connected networks. The model of Equation (8) therefore provides a useful tool for focusing development effort in order to optimize sPyNNaker for different SNN topologies.

**Figure 12:**
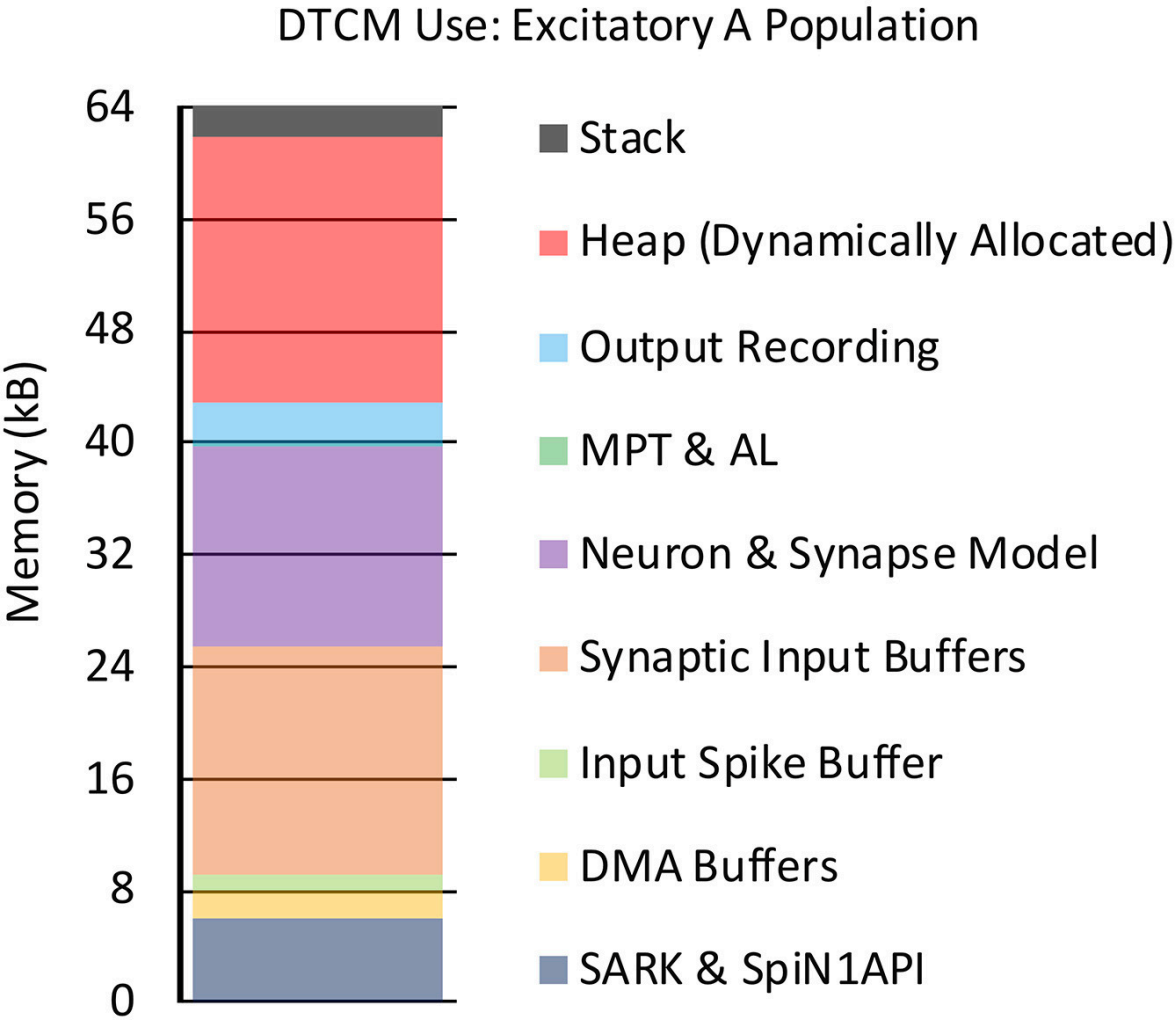
DTCM footprint for neuron application simulating the Excitatory A population in Figure 3B.

In addition to core processing, memory allocation is also an important consideration for a SpiNNaker application. To demonstrate typical memory consumption, the use of DTCM by the application simulating the Excitatory A population in Figure 3B is shown in Figure 12 [explanations of memory use are detailed in section S1.4 (Supplementary Material)]. When simulating 255 neurons per core, the synaptic input buffers require 16.32kB (section 3.2.2), while the current-based LIF neuron and synapse model parameters require 14.28kB. The data structures for locating synaptic information associated with an incoming spike (section 3.2.4), require 0.084kB to define entries covering 5 source vertices, one with two *address list* rows. Additional space is required for the DMA and input spike buffers, and for recording of output state variables. The remaining space is occupied by the operating system libraries SARK and SpiN1API (≈6kB), stack (2kB), with the rest allocated as heap.

The large heap appears wasteful for this example network, however when an SNN contains more populations and projections, a vertex is likely to receive spikes from many different locations, and hence the *master population table* length and number of *address list* rows will increase significantly requiring additional memory. It is also worth noting that additional memory can be allocated to improve runtime performance. For example, synaptic matrices associated with high frequency sources can be stored in DTCM, negating their transfer from SDRAM on spike arrival.

## 5. Discussion

This work presents the latest version of sPyNNaker: a software package for running PyNN SNN simulations on the SpiNNaker neuromorphic platform. It provides a detailed overview of software executed on the SpiNNaker machine, and shows how this interacts with the underlying hardware to achieve realtime execution. An event-driven operating system is described, together with details of the individual callbacks facilitating realtime neural simulation. Neuron updates are time driven, while spike processing is event driven, and handled by a series of pipelined callbacks to optimize performance. Implementation of standard neuron models from the literature is then discussed, both from the perspective of software engineering, and the performance impact of different numerical modeling techniques. Results are then presented for an example network used as a vehicle for explanation throughout the methods section, demonstrating realtime execution of a balanced oscillating SNN. Finally, performance analysis of key operations is presented, demonstrating how neuron and spike processing interact at runtime to govern the limits of realtime simulation.

This software aims to address the original design goal of the SpiNNaker hardware: to simulate large-scale SNNs in realtime. Neurons were intended to be simulated 1,000 per core, and to have 1,000 incoming connections, switching at 10Hz: giving a potential 10,000 synaptic events per neuron per second (10 per neuron per ms, and 10,000 per core per ms). Neuron updates were to be time driven with a default simulation step size of Δ*t* = 1ms, giving 200,000 clock cycles between neuron updates to achieve realtime execution. A number of features have deviated from the original design, including: the use of C code and an event-based operating system to program cores, where it was originally intended to use assembly code and interrupt service routines to define neuron behavior; and the reduction of the default number of neurons simulated by a core to 255. To quantify the impact of these design changes, performance of the presented software is assessed in terms of the maximum number of synaptic events which can be processed while maintaining realtime simulation. Performance is dependent on SNN topology and how the simulation is partitioned onto machine resources (see Figure 11), however over 5,000 synaptic events were handled by cores simulating postsynaptic populations of 255 neurons in fully-connected SNNs—demonstrating performance within a factor of two of the original design target.

However, SNN topology plays an important role in the total number of synaptic events which can be processed within a timestep, as spikes which target greater numbers of postsynaptic neurons enable the fixed costs associated with spike processing to be amortized over more synaptic events. To this end, increasing the number of neurons simulated by a core improves the total number of synaptic events which can be processed, however this effect peaks around 255 neurons per core for the majority of connection probabilities, as the increased time the core must spend updating neurons leaves less for spike processing. This variation in performance with the number of neurons simulated on a core is counter intuitive, as one would expect reducing the number to help parallelize computation and reduce the load on a single core. However it is seen for all cases except a fully-connected network, that the total number of synaptic events which can be processed reduces with neurons per core for connection probabilities of <50% (see Figure 11B). The reduced default of 255 neurons per core is therefore a sensible design choice, as it is close to maximizing the peak number of synaptic events which can be processed for all connection probabilities.

To characterize system performance a detailed profiling analysis was performed (see section S1 in Supplementary Material), resulting in the cost model in Equation (8). This model revealed three SNN-independent parameters governing performance: *m*_*n*_ the time taken to update a single neuron; *m*_*s, s*_ the time taken to evaluate the synaptic contribution of a single spike to a single postsynaptic neuron; and *c*_*s, s*_ the fixed cost associated with an incoming spike in the active spike processing pipeline. To reduce *m*_*n*_ would require more efficient handling of the neuron state update, but also the infrastructure code managing the circular synaptic input buffers, and the shaping of synaptic currents. The value of *m*_*s, s*_ is already close to the original design target of 20 instructions per spike event, and at 23clock cycles is near the optimum which can be produced by standard compilers. However, given the repeated operations performed while processing the synaptic row, there is potential to use hand-crafted assembly code to interleave operations and reduce total row processing times. There is greater scope for minimizing the fixed cost of processing a spike in the pipeline *c*_*s, s*_. For example, while the *master population table* search is relatively efficient, the surrounding infrastructure software could be improved significantly. There is also potential to remove the use of interrupts and events to cycle between the functionality accessed through repeated calls to _dma_complete_callback, and hence avoid the cost of context switching. This means a reduction of 50% is viable for *c*_*s, s*_, and if this could also be achieved for *m*_*n*_ and *m*_*s, s*_, then Equation (8) predicts the original design target of 10,000 synaptic events per ms would be achievable. Simulating 255 neurons per core, these reductions would enable ≈12,700 synaptic events for a fully-connected projection, ≈10, 250 at 30% connectivity, and ≈4, 050 at 5% connectivity.

This work presents a comprehensive overview of the sPyNNaker software package and assesses its performance. It is noted that alternative prototype software models have also been investigated by the SpiNNaker group: such as distributing computation for a group of neurons across multiple cores, each with bespoke roles (Galluppi et al., 2015; Knight and Furber, 2016). This research, together with this work, demonstrates the continued potential of the SpiNNaker chip, and provides important information to inform the design of the next-generation SpiNNaker 2 system.

## Author contributions

This work presents the latest version of the sPyNNaker software package produced by the SpiNNaker group at the University of Manchester, UK. The current SpiNNaker software team is comprised of CB, DF, AG, OR, and AS, and led by AR, all of whom have made significant contributions to sPyNNaker. SD, DL, and LP are researchers within the SpiNNaker group, and worked on earlier versions of SpiNNaker software, and provided assistance with low-level programming and hardware interactions during performance analysis. MM assisted with performance profiling, and PB with tuning and development of the example random balanced network used throughout. OR led the research and wrote the manuscript, while SF leads the SpiNNaker project and supervised this work. All authors reviewed and refined the final manuscript.

### Conflict of interest statement

The authors declare that the research was conducted in the absence of any commercial or financial relationships that could be construed as a potential conflict of interest.

## Footnotes

^1^https://github.com/SpiNNakerManchester/sPyNNaker

^2^https://www.iso.org/obp/ui/#iso:std:iso-iec:tr:18037:ed-2:v1:en

^3^A detailed guide to this process can be found at: http://spinnakermanchester.github.io/workshops/seventh.html
