# Supplementary material for: sPyNNaker: A Software Package for Running PyNN Simulations on SpiNNaker
Source: Front Neurosci. 2018 Nov 20;12:816. doi: 10.3389/fnins.2018.00816 (PMC6257411; doi:10.3389/fnins.2018.00816)
Supplement: Supplementary file 1 [file Data_Sheet_1.PDF]

Supplementary Material: sPyNNaker: a Software Package for Running PyNN Simulations on SpiNNaker

1 PERFORMANCE PROFILING

To evaluate execution performance of the sPyNNaker software, the event-based callbacks are profiled to measure the amount of time spent in core functions, and the associated impact on SNN performance. This section begins with assessment of the processing overheads of individual callbacks, and then demonstrates how they combine at runtime. Holistic cost models are then developed to predict the capacity of a given core when simulating an SNN containing varying levels of connectivity. Finally, the memory footprint of a typical core is assessed and reported.

To measure elapsed time, two readings are taken from the free-running clock on each SpiNNaker core, with each reading stored in an array in DTCM. On simultation completion, the difference between associated values is calculated to give a timing measurement in clock cycles (cc). The cost of the first clock read and store is included in this measurement, and is therefore subtracted from all measured results to get the true elapsed time. This cost is measured at $5\,$cc, where at $200\,$MHz the period of a single clock cycle is $5\,$ns. Note that for comparison and consistency reasons, all measurements below are reported in microseconds. Unless otherwise stated, all results are recorded over 100 events, and the minimum, maximum, mean, and standard deviation of all cases reported. Where a test requires multiple cores to utilise SNN functionality, unless otherwise stated, all measurements are performed on a neuron application executed on physical core 2 of a random selection of SpiNNaker chips from the large SpiNNaker machine hosted at the University of Manchester. To aid performance predictions from measurements, a number of cost models are generated from measured data. Due to measuring output from compiled code following repeated execution paths, results are consistent and scale linearly, meaning linear cost models are generated with values of $R^2 = 1$ unless otherwise stated.

1.1 Individual Callback Performance

In the following measurements, the backbone software costs of Spin1API and SARK are typically small relative to those of the callbacks they schedule. However, a significant cost which must be evaluated outside the individual callbacks is that of responding to a hardware interrupt, generating an event, and scheduling the associated callback – see vertical black lines in Fig. 9. To evaluate these costs, interrupts are disabled within a `timer_callback` on a single core running a sPyNNaker application, and each event triggered in isolation. The measurement start time is then read from the clock, and interrupts are enabled causing the core to respond to the event, and the measurement end time is read immediately on entering the associated callback. The difference is then calculated and the cost of enabling interrupts ($14\,$cc) is subtracted yielding the response times in Tab. S1. Response times are callback and priority dependent, with the -1 priority event responding quickest due to it not queuing callbacks, and because the code to be executed is accessed directly via a pointer by the operating system. Conversely, the priority 2 callback is the slowest to respond, which is as expected due to its queuing of callbacks, and the operating system checking for higher priority tasks before dispatching a callback. The two priority 0 events have similar response times, with the software event triggered `user_callback` responding slightly quicker as no hardware event handling is required.

Interrupt Priority	Response to Callback	Time (cc)	Time (μs)
2	`timer_callback`	374	1.87
-1	`_multicast_packet_recevied_callback`	50	0.25
0	`user_callback`	89	0.445
0	`_dma_complete_callback`	121	0.605

	Callback to Callback Transition	Time (cc)	Time (μs)
	`user_callback` to `_dma_complete_callback`	172	0.86
	`_dma_complete_callback` to `_dma_complete_callback`	179	0.86

Table S1. Context switching costs of different events and their associated callbacks

Also included in Tab. S1 are the costs associated with exiting a callback and entering one which is already queued. This is an important consideration in the spike processing pipeline, where in an active pipeline the transition between instances of `_dma_complete_callback` happens frequently, taking $0.86\,\mu$s each time. Together with the interrupt response times, these costs are features of using an event-driven operating system such as SpiN1API, which provides a trade-off between performance and masking the complexity of interacting directly with hardware.

1.1.1 `timer_callback`

The periodic updating of neuron and synapse states consumes a significant proportion of core processing time. This code is critical to overall system performance, as it governs the speed at which the system can run together with the remaining time available to process incoming spikes. Results from execution of a `timer_callback` when simulating both LIF and Izhikevich neurons (with both current- and conductance-based exponential synapses) are detailed in Fig. S1. The data shows mean results per callback, calculated from running 100 simulation timesteps (100 calls to `timer_callback`). Note that no spikes are emitted or received during this test, meaning no additional processing overheads are incurred, and that no refractory dynamics are encountered, and hence the cost of updating a single neuron is fixed. Results are shown for populations containing increasing numbers of neurons, enabling evaluation of per-neuron performance together with fixed processing costs associated with the background neuron handling framework. The LIF neuron is executed at realtime with a simulation timestep of $1\,\text{ms}$, giving a total of $200,000$ instruction cycles between calls to `timer_callback` – however the size of the simulation timestep Δt does not impact the cost of the callback (only numerical accuracy within the simulation). The total callback time for the LIF model increases linearly with the number of neurons, with a fixed processing overhead of $1.365\,\mu$s ($11.781\,\mu$s with full recording), and an additional cost of $1.015\,\mu$s ($1.007\,\mu$s with full recording) per neuron. The cost of processing the Izhikevich neuron with current-based synapse shaping also increases linearly with the number of neurons, with a fixed processing overhead of $1.361\,\mu$s ($11.763\,\mu$s with full recording), and an additional cost of $1.450\,\mu$s ($1.441\,\mu$s with full recording) per neuron. This information is summarised in Tab. S2, including the fixed cost of responding to the *timer* event.

While these costs describe the typical behaviour of a population of sub-threshold neurons, small changes in processing time will occur when an individual neuron emits a spike, or is refractory. On spiking, the neuron must call functions to update its state and begin any refractory dynamics, and must also call a SpiN1API function to send a packet to the router. The additional costs associated with spiking for the neuron models of Sec. 3.3 are detailed in Tab. S3. The additional operations of the Izhikevich neuron model (Eq. 7) require additional processing relative to the LIF neuron. Post spiking, the lack of separate refractory

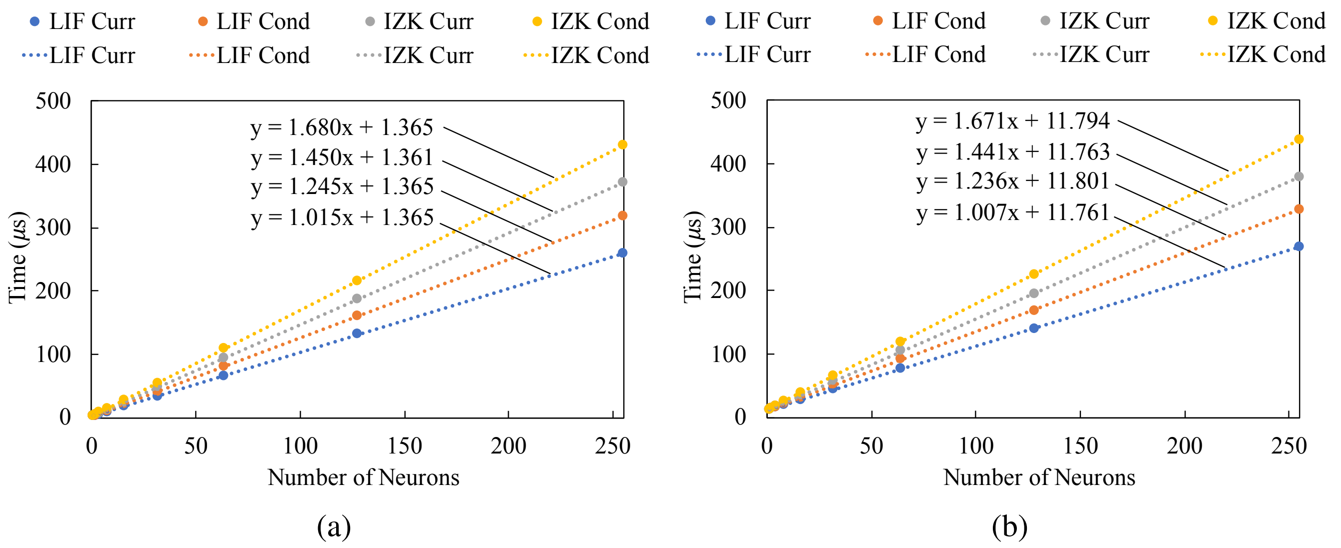

Figure S1: Performance measurements and linear cost models characterising execution time of `timer_callback` for a range of neuron and synapse model combinations, all for a range of numbers of neurons: (a) no output data recording; (b) full output data recording (spikes, V, $g_{syn,exc}$, and $g_{syn,inh}$).

dynamics mean the Izhikevich neuron requires the same sub-threshold level of processing. However, the LIF neuron benefits from a reduced update time during its refractory period due to its clamped membrane potential. When considering a LIF neuron as part of a larger population simulated on a single core, this reduced update time during a refractory period has the benefit of reducing the total population update time, and compensates for other neurons which have spiked during this update (and hence paid the additional cost of spiking). It may also allow the core to catch up if multiple neurons spiked in previous updates and caused the core to lag behind its counterparts.

Nueruon model	Synapse Model	Recording	m_n (μs / neuron)	c_n (μs)
LIF	Current	None	1.015	3.235
LIF	Current	Full	1.007	13.631
LIF	Conductance	None	1.245	3.235
LIF	Conductance	Full	1.236	13.671
Izhikevich	Current	None	1.450	3.231
Izhikevich	Current	Full	1.441	13.633
Izhikevich	Conductance	None	1.680	3.235
Izhikevich	Conductance	Full	1.671	13.664

Table S2. Variable and fixed neuron processing costs, including cost of responding to *timer* events (see Tab. S1).

Neuron Model	Sub-threshold Update Time (μs)	Refractory Update Time (μs)	Additional Cost of Spiking (μs)
LIF	0.405	0.185	0.205
IZK	0.84	0.84	0.3

Table S3. Components of single neuron update times and variations due to firing and refractory period

1.1.2 _multicast_packet_received_callback

On receiving a packet, the core immediately responds with the callback detailed in Sec. 3.2.3. Execution can follow two routes depending on whether the spike processing pipeline is active: if the pipeline is inactive this callback must additionally raise a software event to kick-start the spike processing pipeline (see Sec. 3.2.4). Measurements are taken encapsulating the functions internal to the callback, with zero variability recorded across all tests, as shown in Tab. S4. It is observed that when the pipeline is active this callback is $\approx 2\times$ faster than when inactive.

Scenario	Time (μs)
Pipeline inactive	0.44
Pipeline active	0.23

Table S4. Cost models for execution of `_multicast_packet_received_callback`

1.1.3 user_callback

The `user_callback` responds to the *user* event, and kickstarts the spike processing pipeline. The callback calls the function `setup_synaptic_dma_read`, which uses the latest key in the spike input buffer to locate and copy the associated synaptic data from SDRAM to core DTCM. This function is the same as that called from within the `_dma_complete_callback` to initiate processing of the next existing spike and sustain the spike processing pipeline. Its performance within the `user_callback` is reported here, but should also be used when predicting costs of `setup_synaptic_dma_read` executed from within a `_dma_complete_callback`.

The function `setup_synaptic_dma_read` first retrieves the next spike ID to process from the input spike buffer, and uses it to perform a binary search of the *Master Population Table*. The cost of searching this table for a variety of search depths (i.e. numbers of source vertices) is shown in Fig. S2(a), with the best and worst case search times for a source vertex in each layer reported in (b). This data was recorded from an SNN containing a single target neuron executed on a single core, with variable numbers of spike sources each executed on a single vertex. Each spike source is timed to send a single spike to the target neuron at a unique time. This generates a *master population table* on the target neuron vertex of length equal to the number of source vertices, and causes lookup of each item exactly once. Timing measurements recorded from the search, are displayed in Fig. S2 – no variation is observed across repeated measurements. The binary search algorithm proves efficient for searching the structured *master population table*, with per-layer worst and best case search times increasing linearly with search depth. When formulating a machine graph and creating data structures for a sPyNNaker application, no assumptions are made about network activity, and hence the *master population table* structure is not optimised to return entries from a particular source. When developing a cost model for this search time, it is assumed that spikes will be received at the same rate from all source vertices in the table. Due to small implementation differences in searching the upper and lower half of a search region, the worst case search time for an individual layer increases at a higher rate than the best. However, from Fig.S2(a) it is seen that this has a limited effect on search times within a layer, and an average of these two extremes provides a conservative estimate of the average layer search time (dashed red line).

To understand the contribution of this search to `setup_synaptic_dma_read`, total function execution time is measured within an SNN simulation. A single spike source neuron, and single target neuron population are connected via a single projection, and individual spikes emitted from the source at

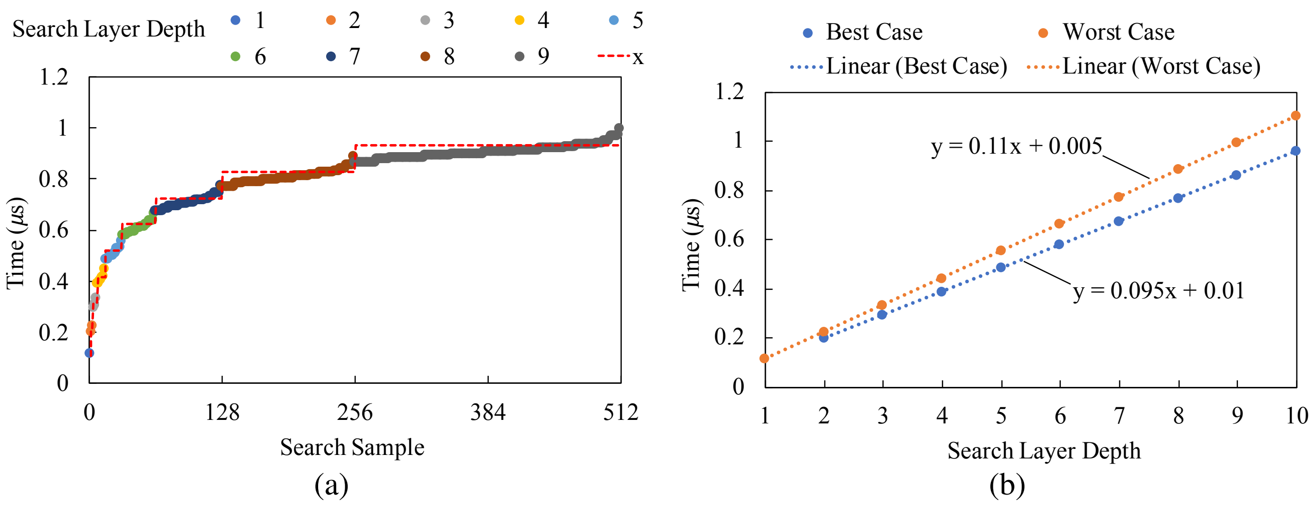

Figure S2: *Master population table* lookup time with search depth. Left: lookup for increasing numbers of source vertices; Right: measurements of best $(0.11x + 0.005)$ and worst $(0.095x + 0.01)$ case search times within a search layer, together with cost models (dashed lines).

unique times. Each spike therefore requires kick-starting of the pipeline, triggering execution of the `user_callback`. Measurement of the callback contents, which comprise a single call to the function `setup_synaptic_dma_read` are reported in Tab. S5. These measurements show the contribution of the single-layer *master population table* search to the total callback time is relatively small, with the fixed costs associated with DMA setup and the search framework dominating. However, with a fan-in from over 512 vertices, the worst-case contribution will increase by a factor of ≈ 10, making a significant contribution to total callback time.

1.1.4 `_dma_complete_callback`

On DMA completion, the synaptic row is in DTCM and must be processed to convert each synaptic word into neural input. To measure the cost of this processing, readings from the clock are taken on entering and exiting `_dma_complete_callback`. A test SNN is created with a spike source population containing a single spike source neuron, connected via an all-to-all connector to a single target population of LIF neurons of variable number. Spikes from the single source are timed to arrive at the postsynaptic neuron in the window between calls to `timer_callback`, after the preceding call has completed: therefore all overheads reported in Fig. S3 are a direct result of row processing only. Each test is independent of the neuron model, and does not include synaptic plasticity. A range of synaptic row lengths is tested, corresponding to the fan-out from a presynaptic neuron to a range of postsynaptic neurons, and a linear relationship is observed between number of synaptic words and processing time.

At the beginning of the callback, in order to sustain the spike processing pipeline, a single function call to `setup_synaptic_dma_read` is made (the same function called from within `user_callback`. This aims to setup the DMA for the next synaptic row to process, however when called within the active spike processing pipeline there are now multiple execution paths. In addition to searching the *master population table* with a new key from the input spike buffer, it is possible that a subsequent *Address List* row must be processed for the current spike (such as when the core simulating Excitatory A in Fig. 3(b) receives a spike from one of its neurons – see Fig. 7), or that the input spike buffer is empty and the pipeline should be deactivated. Timing data for these alternative execution paths is presented in Tab. S5.

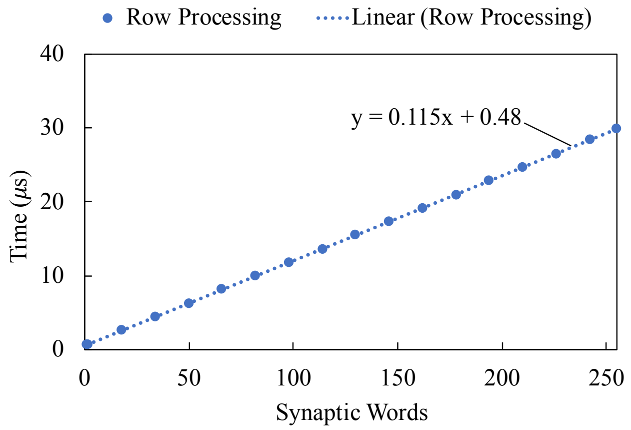

Figure S3: Processing synaptic row into neuron input: measured data (markers) and cost model demonstrating a fixed overhead of $0.480\,\mu$s, with an additional cost of $0.115\,\mu$s per synaptic word.

Execution Paths of `setup_synaptic_dma_read` when called from `_dma_complete_callback`	Time (μs)
`_dma_complete_callback` prior to DMA request	1.25
Check there are no further rows for this MPT entry, and lookup next spike	2.14
Search for next spike but the input spike buffer is empty, so deactivate pipeline	0.66
Accessing subsequent row on same MPT entry	1.472

Table S5. Processing times from a range of execution paths through the function `setup_synaptic_dma_read`, which is called from both the `user_callback` and `_dma_complete_callback` to initiate DMA transfer of the next synaptic row to process.

1.2 DMA Performance

Performance of a direct memory access (DMA) is an important consideration in overall spike processing performance. An overview of execution costs is provided here, both in terms of direct performance, and in the context of a sPyNNaker application. Profiling is then extended to explore performance variations between isolated individual cores, and when multiple cores contend for SDRAM data.

1.2.1 sPyNNaker DMA Performance

To isolate outright DMA performance from the SpiN1API event-based operating system, timing measurements are taken around the DMA request within the `user_callback`. In order to stop the callback continuing to completion and the system following the remainder of the spike processing pipeline, immediately after completing a DMA request, the DMA controller is polled within a while loop to check DMA state, and on DMA completion the final timing measurement is made. Total transfer time is reported for a range of data sizes in Fig. S4(a). Total DMA time is approximately linear with the size of transferred data, with slight variations introduced from bursting operation and the interaction between the core DMA and SDRAM controllers. A uniform standard deviation is observed with range of $\approx 4\,$cc, which increases linearly with data size, giving a variation of $\approx 10\,$cc ($\approx 50\,$ns) when transferring data representing the maximum size of a synaptic row (258 words). The fixed cost of $0.897\,\mu$s is introduced by the software function calls setting up the DMA.

Figure S4(b) shows the same test, performed within the typical sPyNNaker spike processing pipeline. Now the `user_callback` is free to return on callback completion, and the final timing measurement is made immediately on entering the `_dma_complete_callback`. While the per-word transfer cost

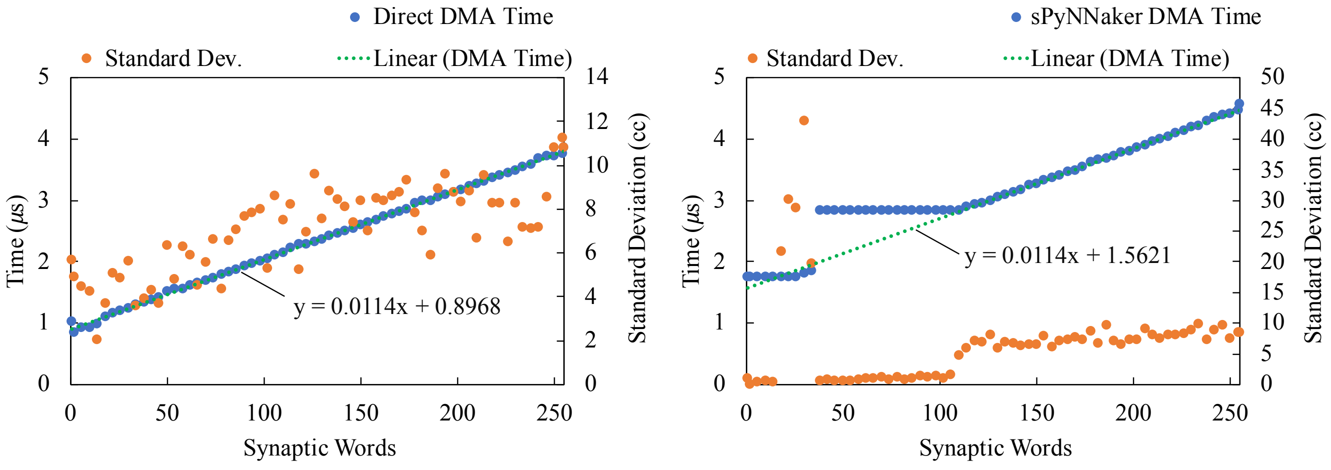

(a) (b)

Figure S4: Minimum DMA transfer time measured outside typical neuron processing conditions: over a 100 trials a linear cost model estimates a fixed cost of $0.897\,\mu$s, plus 0.011μs per synaptic word. Costs include overhead of software function calls to setup DMA. (a) Direct DMA time, recorded outside event-based operation; (b) DMA time as recorded within a sPyNNaker application, measuring time from function call requesting DMA to entering `_dma_complete_callback`.

remains the same ($0.011\,\mu$s), several fixed processing costs are introduced. For small data sizes (< 40 synaptic words), the DMA completes before the core exits the `user_callback`, meaning a *DMA complete* event is registered and waiting on exit, and SpiN1API immediately schedules and begins execution of `_dma_complete_callback`, resulting in a fixed cost of $1.745\,\mu$s. For transfers between 40 and 105 synaptic words, the DMA completes after the `user_callback` has exited, and the core returns to examine the callback queues within SpiN1API. To avoid contention interrupts are disabled during this process, meaning if the DMA completes during this time the core cannot immediately respond to the associated event. Instead, it can only do so after interrupts are re-enabled, leading to a constant response time of $2.85\,\mu$s. The standard deviations of these measurements are ≈ 0, as expected behind fixed operating system costs. For large data transfers (> 105 synaptic words), transfer time exhibits the response (and variation) shown in Fig. S4(a), albeit with a larger fixed component due to addition of the context switching cost of responding to the *DMA complete* event. Note, that the behaviour of a DMA of $38 <$ synaptic words < 105 is modified if the core was already processing a callback prior to processing the spike, such as a lower priority `timer_callback`. Under these circumstances, the core will return to processing of the `timer_callback` on completion of the `user_callback`, meaning the core will respond immediately to the subsequent *DMA complete* event, and the response time follows the behaviour of > 105 synaptic words.

1.2.2 DMA Core to Core Variation

Due to chip layout, and cores having different paths to the SDRAM controller, variation is expected between cores when performing the same task in isolation across different cores. Figure S5 shows the total DMA time (as measured in Fig. S4(a)) for a range of data sizes. The average transfer time is reported by the coloured marker, while upper and lower error bars denote the maximum and minimum measurements respectively. In all cases it is seen that the average measurement is close to the minimum, with outliers defining the maximum. Note that core 10 is the chip monitor core in this test, meaning it is not possible to

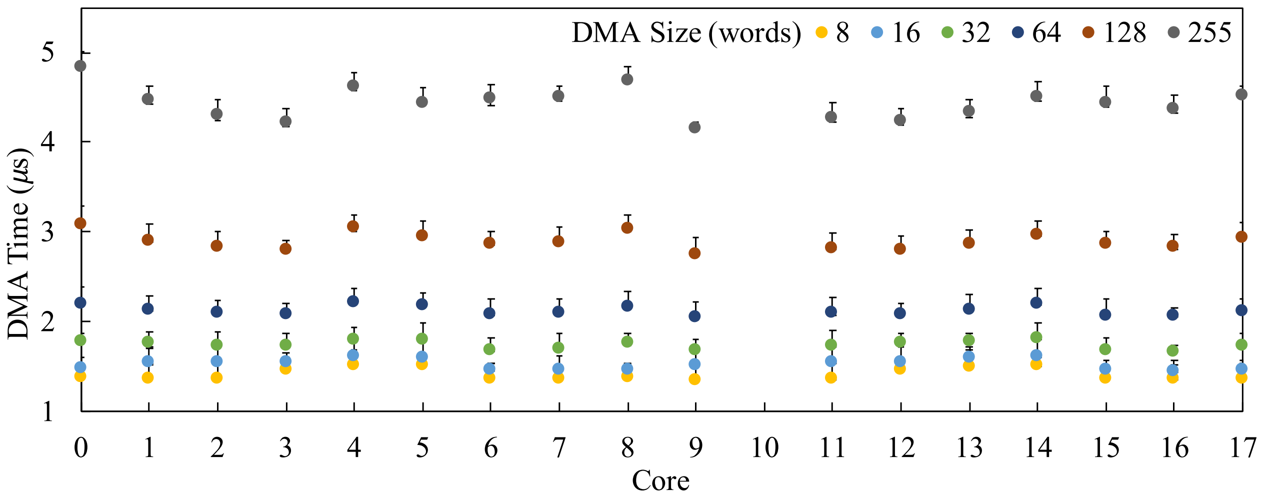

Figure S5: Comparison of isolated DMA performance for each core on a single chip (core 10 is the chip monitor).

test its DMA performance. While small variations exist between cores, this test shows there is relatively little variation, enabling cost models to be built from the data collected above.

1.2.3 DMA Contention

In a large simulation, multiple cores on a the same chip will run neuron modelling applications. These applications may well be modelling multiple sub-populations for the same parent population, and hence will likely share PyNN level projections. This in turn means that multiple cores will receive packets representing the same spike simultaneously. While this is not an issue for the asynchronously operating cores, it can impact DMA performance as multiple cores will simultaneously request synaptic data associated with a spike. Transfer times will increase due to the finite SDRAM bandwidth, and from latency introduced from sequential processing of DMA burst requests by the SDRAM controller. To demonstrate the effect of SDRAM DMA contention, a test application is compiled which performs 1000 repeated DMA transfers for a range of data sizes and measures total transfer time as in Sec. S1.2.1. This application is then executed on multiple cores simultaneously to measure the effect of contention. Maximum DMA time for each scenario is plotted in Fig. S6 for physical core 0 (representing the worst case when core 10 is the monitor as shown in Fig. S5).

For DMAs of < 25 words, no degradation in maximum DMA time is observed, as requested data can be transferred in a single burst, and over the repeated tests core 0 ends up at the back of the queue on the SDRAM controller at least once. The transition between 2 and 4 contending cores sees the DMA read bandwidth of $600\,\mathrm{MBs}^{-1}$ saturate for larger data sizes. When 8 cores and above simultaneously request data, performance is reduced significantly: maximum DMA time is increased by $\approx 2\times$ for transfers of 125 words, and $\approx 2.6\times$ for transfers of 250 words. This effect is compounded as more cores contend, and when 16 cores request data simultaneously maximum DMA time increases by $\approx 4.5\times$ and $\approx 5.4\times$ for 125 and 250 word transfers respectively.

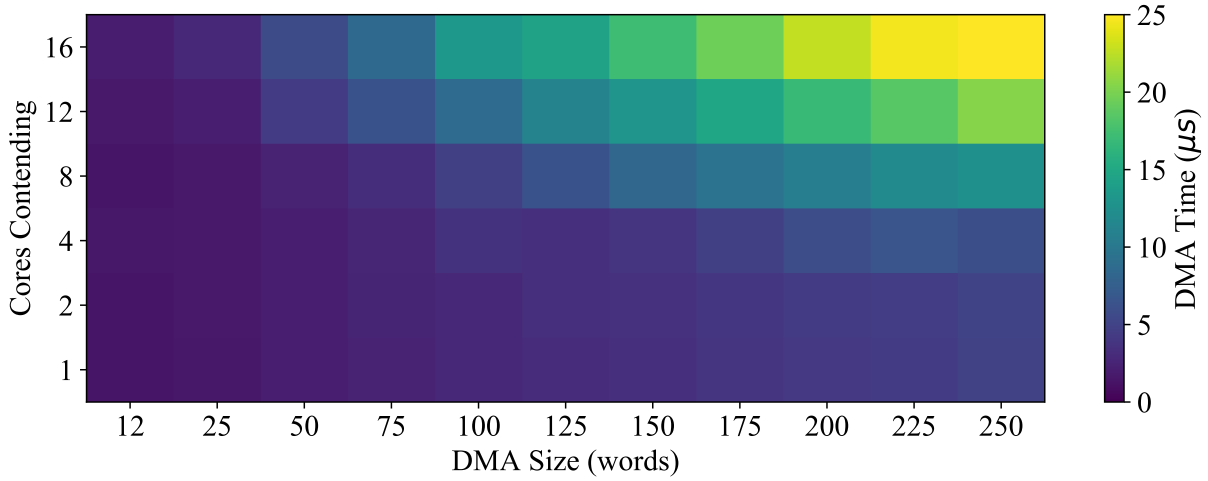

Figure S6: Total DMA time for a range of data sizes from the perspective of core 0, when additional cores are simultaneously requesting data from SDRAM.

1.3 Callback Interaction

The individual cost measurements reported above can be combined to predict the total cost of processing both: a single incoming single spike; and the arrival of multiple spikes and their handling via the spike processing pipeline.

1.3.1 Processing a Single Spike

To predict the cost of processing a single spike requires consideration of the interaction between multiple callbacks as shown in Fig. S7(a). From the individual measurements and linear cost models developed in Secs. S1.1 & S1.2, it is possible to superpose fixed processing costs which must be paid once per spike, and variable costs which are paid once for every neuron a spike targets. This in turn leads to the total spike processing cost model presented in Tab. S6. Three regimes are presented for the different possible

Variable Contributions (m_s)		Cost (μs / synaptic word)	
Row Processing	0.115	0.115	0.115
DMA Fetch	0	0	0.011
**Total**	**0.115**	**0.115**	**0.126**

Fixed Contributions (Single Spike) (c_s)		Cost μ(s)	
`_multicast_packet_received_callback`	0.44	0.44	0.44
`user_callback` (prior to DMA request)	1.25	1.25	1.25
Fixed DMA cost (see Fig. S4(b))	1.745	2.84	1.562
`setup_synaptic_dma_read` from within `_dma_complete_callback` (deactivates pipeline)	0.66	0.66	0.66
Process Row	0.48	0.48	0.48
Context switching (from *user* event, *DMA complete* event context switch included in fixed DMA costs)	0.445	0.445	0.445
**Total**	**5.02**	**6.11**	**4.837**

Table S6. Single spike processing costs for the process shown in Fig. S7(a)

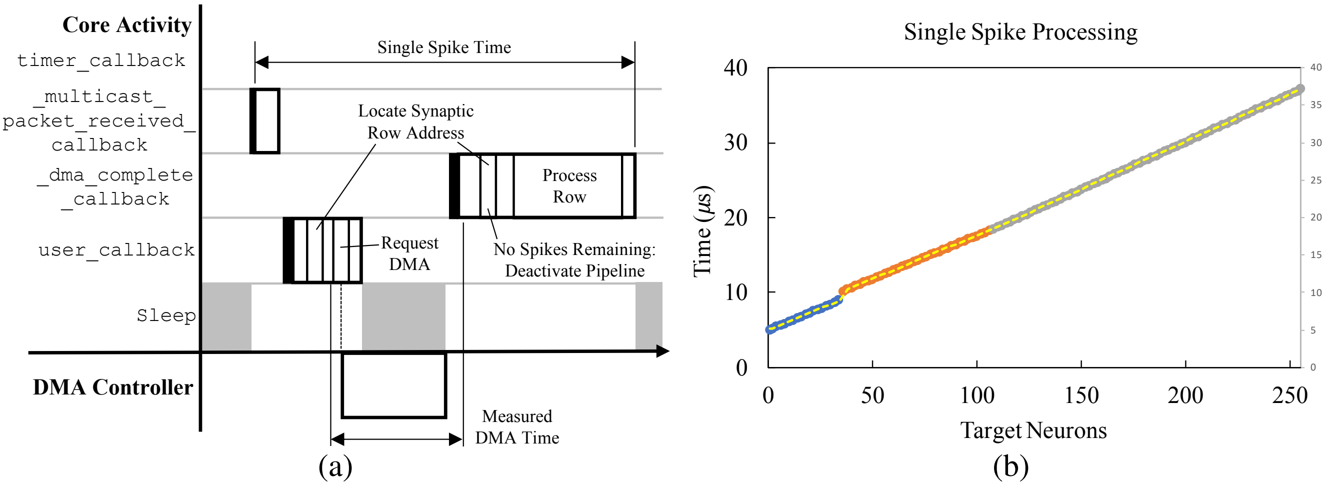

Figure S7: (a) Schematic detailing components of a single incoming spike, and (b) spike processing costs measured for simulating a spike targetting increasing numbers of postsynaptic neurons, together with cost model prediction (dashed yellow line).

responses of the DMA based on transfer size. When operating transfers below 105 synaptic words the DMA completes before the `user_callback` exits, or while the operating system has disabled interrupts, meaning there is no DMA-based contribution to the variable component of spike processing, and the fixed contributions are associated with those shown in Fig. S4(b). Additional context switching costs come from responding to the *user* event and entering the associated callback, while fixed contributions are made from processing the synaptic row, executing the `_multicast_packet_received_callback`, and the second call to `setup_synaptic_dma_read` within the `_dma_complete_callback`, which finds no further spikes to process and deactivates the spike processing pipeline. The time required to process a single spike t_s, is therefore given by Eq. S1.

$$t_s = m_s n + c_s \quad \text{where} \begin{cases} m_s = 0.115,\ c_s = 5.020 & \text{for} \quad n < 45 \\ m_s = 0.115,\ c_s = 6.110 & \text{for} \quad 45 < n < 105 \\ m_s = 0.126,\ c_s = 4.837 & \text{for} \quad 105 < n \end{cases} \tag{S1}$$

To corroborate these predictions, the end-to-end processing time of a single spike (t_s), is measured according to Fig. S7(a) for varying numbers of target neurons. Measurements begin on entering `_multicast_packet_received_callback`, and end directly before exiting `_dma_complete_callback`. Predictions from Eq. S1 are displayed in Fig. S7(b), showing good agreement with measurements taken directly from simulations. While treating the three regimes of the DMA processing individually produces an accurate model for spike time prediction, it is observed that extending the model capturing the region for > 105 synaptic words down to 0 provides a good approximation, and hence models make use of this assumption when predicting pipeline performance in Sec. S1.3.2.

1.3.2 Processing Multiple Pipelined Spikes

Following the successful profiling of a single spike, the same approach is applied to the spike processing pipeline, with the aim of characterising how many spikes can be processed within a set period of time. Pipeline operation is demonstrated by the processing of spikes 2, 3, and 4 in Fig. 9, and is characterised by three different types of spike: the *first* spike in the pipeline, multiple *subsequent* spikes, and the *last* spike

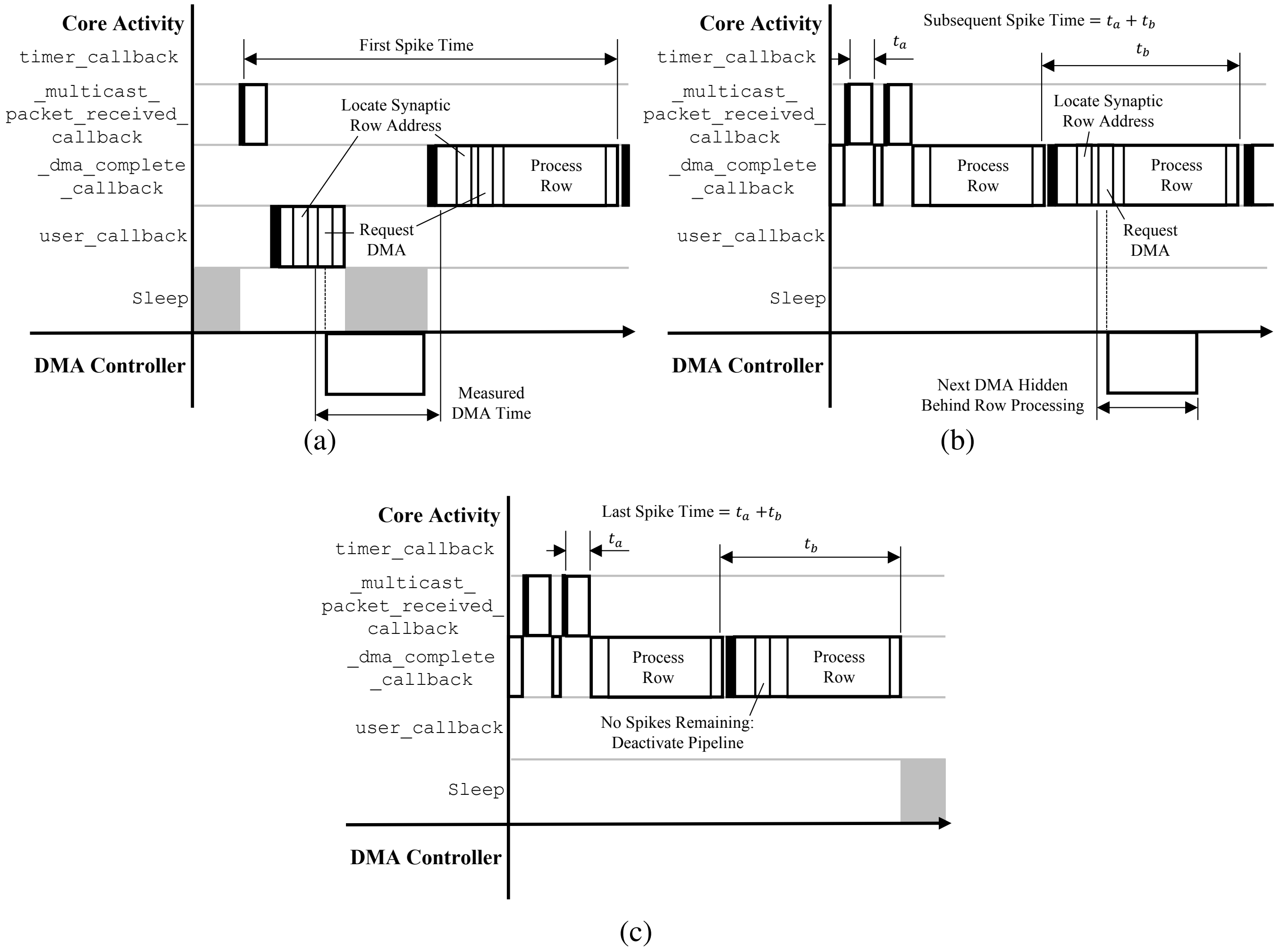

Figure S8: Schematics detailing components of spike processing pipeline: (a) first spike, (b) subsequent spikes, and (c) last spike.

in the pipeline – as shown in Fig. S8. These distinctions are made due to the different actions of each spike type in relation to the spike processing pipeline. Processing of the first packet to arrive at the core must activate the spike processing pipeline, register a *user* event to locate the address of the associated synaptic information, and initiate a DMA to bring it into core DTCM (see Fig. S8(a)). If further packets are received by the core during this process, then the associated spikes will be processed in the activated pipeline. On entering the `_dma_complete_callback` of the first spike, the synaptic row address associated with the subsequent spike is located and a DMA initiated. The core then continues to process the synaptic row of the first spike, converting each synaptic word into the appropriate synaptic input buffer contribution. On completion of the `_dma_complete_callback`, processing of the first spike is complete, and the `_dma_complete_callback` associated with the subsequent spike is begun (see Fig. S8(b)). This process repeats for each subsequent spike, however when processing the `_dma_complete_callback` for the last spike, there are no further spikes to be processed, and hence no DMA is performed and the pipeline is deactivated (see Fig. S8(c)).

It is possible to characterise each type of spike according to the scenarios shown schematically in Fig. S8. The data used in this characterisation process is displayed for the three spike types in Tab. S7.

Variable Contributions (μs / synaptic word)	$m_{s,f}$	$m_{s,s}$	$m_{s,l}$
DMA transfer	0.011	0	0
Process Row	0.115	0.115	0.115
**Total**	**0.126**	**0.115**	**0.115**

Fixed Contributions μ(s)	$(c_{s,f})$	$(c_{s,s})$	$(c_{s,l})$
`_multicast_packet_received_callback`	0.44	0.23	0.23
`user_callback` (prior to DMA request)	1.25	N/A	N/A
DMA Fixed costs	1.562	0	0
`setup_synaptic_dma_read` from within `_dma_complete_callback`	2.14	2.14	0.66
Process Row	0.48	0.48	0.48
Context switching	0.695	1.11	1.11
**Total**	**6.567**	**3.960**	**2.48**

Table S7. Variable and fixed spike processing costs for the: first, subsequent and last spikes in an active spike processing pipeline.

As with the single spike, the first spike in the pipeline must pay a variable cost for both the DMA transfer and row processing. However, because the DMA is hidden behind row processing for both the subsequent and last spikes, their variable components are set based entirely on the variable row processing cost. The first spike spends longer in the `_multicast_packet_received_callback`, as this is where the pipeline is activated. It also incurs the additional overheads associated with the *user* event. The subsequent and last spikes are masked from the DMA fixed processing costs within the `_dma_complete_callback`, and instead experience only the cost of the SpiN1API software call to initiate a DMA, which is included in the `setup_synaptic_dma_read` contribution of $2.14\,\mu$s. The last spike pays a reduced cost here, as there are no further spikes to process, and hence no DMA is initiated and the pipeline is deactivated. Regarding context switching, the first spike includes the *packet received* and *user* events, while the *DMA complete* event overhead is incorporated into the DMA fixed costs. The subsequent and last spikes pay the *packet recevied* event overhead, together with the `_dma_complete_callback` to `_dma_complete_callback` cost from Tab. S1.

The range of coefficients for m_s and c_s in Tab. S7, characterise spike processing and enable bottom-up predictions of the total number of spikes which can be processed in a given period of time. In order to assess realtime performance, it is interesting to set this period of time (t_p) to that between *timer* events (see Sec. 3.2). A combined cost model is developed in Eq. S2, which characterises the number of spikes which can be processed by a neuron application core, after it has completed updating the state of its neurons. The time taken to perform the `timer_callback` is calculated from m_n and c_n in Tab. S2, and then subtracted from the total time between *timer* events (t_p), giving the remaining time available for spike processing. The time taken to process the first and final spikes is then subtracted from this remainder (dealt with in isolation as these spikes follow the paths outlined above – activating and deactivating the spike processing pipeline – with 2 added to the total number of processed spikes). The total remaining time is then divided by the cost of processing a single subsequent spike, in order to evaluate the total number of spikes which can be processed.

$$T_s = \frac{t_p - (m_n n + c_n) - (m_{s,1} nP + c_{s,1}) - (m_{s,l} nP + c_{s,l})}{m_{s,s} nP + c_{s,s}} + 2 \tag{S2}$$

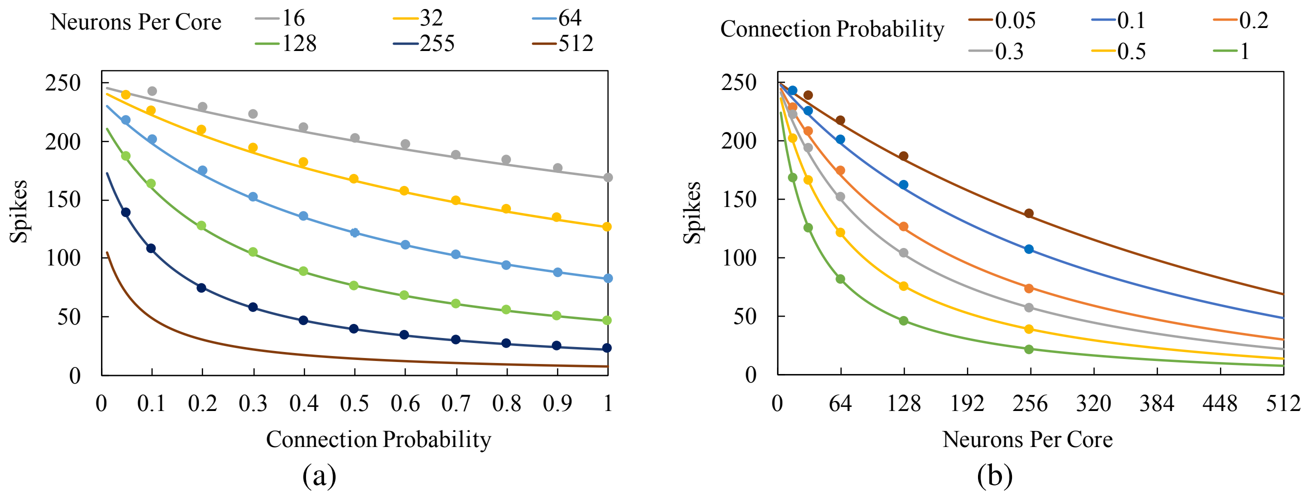

Figure S9: Model predictions for numbers of spikes which can be processed between timer events for a range of connection probabilities and numbers of neurons simulated per core. Markers indicate limiting cases measured directly from sPyNNaker simulations.

Predictions using Eq. S2 and coefficients from Tabs. S2 & S7 are displayed in Fig. S9, for a range of population sizes and connection probabilities. To corroborate these predictions, a corresponding SNN network is simulated via sPyNNaker, containing a single spike source population projecting with variable probability to a target neuron population of variable size. The number of spikes sent between two *timer* events is then increased to find the limiting case where no further spikes can be processed without delaying the subsequent `timer_callback`. Model predictions are in good agreement with measurements, validating the use of Eq. S2 to characterise system performance.

1.4 Memory Use

As well as core processing, TCM memory consumption plays an important part in application performance. For example, available memory impacts the number of neurons which can be simulated on a core, and storage of direct synaptic matrices in DTCM negates the need for a DMA during spike processing. Tab. S8 details the typical DTCM footprint of neuron and spike processing data structures for the core simulating the Excitatory A population containing 255 LIF neurons in Fig. 3(b). The largest datastructures

Item	Cost Model	Typical (bytes)
Stack	2048	2048
Heap	Dynamically allocated from remaining memory	19072
Output Recording	36 (spikes) + $(3 * 1024\,(V,\ g_{syn(exc)},\ g_{syn(inh)}))$	3108
Master Population Table	$(96/8) \times n_{\text{num source vertices}}(5)$	60
Address list	$(32/8) \times n_{\text{address list rows}}(6)$	24
Neuron and Synapse Model	$(32/8) \times n_{\text{params}}(\text{LIF} = 8 + 6) \times n_{\text{neurons}}(250)$	14280
Synaptic Input Buffers	$(16/8) \times n_{\text{receptors}}(2) \times n_{\text{slots}}(16) \times n_{\text{neurons}}(250)$	16320
Input Spike Buffer	$(32/8) \times 256$	1024
DMA Buffers	$(32/8) \times 2 \times (n_{\text{neurons}}(255) + n_{\text{row header words}}(3))$	2064
SARK & SpiN1API	3000 + 3000	6000

Table S8. DTCM use for a core simulating the Excitatory A population in Fig. 3(b): containing 250 LIF neurons and fan-in from 5 source vertices (including itself).

are the synaptic input buffers and neuron and synapse model parameters, which consume approximately half of DTCM. These structures are large due to their containing instances for each individual neuron. The example SNN contains relatively few populations of neurons, meaning the fan-in from source vertices is relatively low, requiring only $0.086\,$kB of memory required to define the *master population table* and *address list*. It is worth noting that for larger networks populations could receive projects from over 1000 source vertices, greatly increasing the size of these data-structures. A total of $3.108\,$kB is allocated to record neuron state variables: a 36 byte bit field to record output spikes, and $1024\,$bytes to record each of membrane potential, and excitatory and inhibitory conductances. Approximately $6\,$kB is used for the event-based operating system, with further memory consumed by buffers for storing incoming spike IDs $(1\,$kB), and enabling DMA transfer of data to SDRAM $(2\,$kB). Finally, stack is allocated $2\,$kB, leaving $\approx 19\,$kB for heap.

2 CALLBACK PSEUDO CODE

Algorithm 1 `timer_callback`

// Update Synapse State
Disable interrupts (stop newly arriving spikes interfering with synaptic input buffers);
for each neuron N on the core **do**
Update existing excitatory synaptic input to time $t + \Delta t$;
Update existing inhibitory synaptic input to time $t + \Delta t$;
for each synapse type s **do**
Get integer sum of weights from synaptic input buffer: indexed by $[n,\ s,\ t + \Delta t]$;
Convert integer sum of weights to fixed-point type (*accum*);
Add contribution to synaptic input;
end for
end for
Restore interrupts;

// Update Neuron State
for each neuron n on the core **do**
Get neuron model components (*neuron model*, *synapse type*, *input type*, *additional input* and *threshold type*);
Get excitatory input from *synapse type* component;
Get inhibitory input from *synapse type* component;
Convert synaptic input to neuron input (based on *input type* component);
Evaluate intrinsic currents from *additional input* component
Update neuron membrane potential based on existing state and new input currents (*neuron model* component);
Store updated membrane potential and synaptic inputs for recording;
if membrane potential is above threshold (*threshold type* component) **then**
Reset membrane potential and set refractory period timer;
Notify *additional input* of spike event;
Add post-synaptic event to memory (for use in plasticity update);
Instruct router to emit spike from neuron N;
end if
Disable interrupts;
Record requested output: spikes, conductances, membrane potential;
Enable interrupts;
end for

Algorithm 2 `_multicast_packet_received_callback`

// Process Multicast Packet
Extract 32-bit integer source neuron ID from packet and add to input spike buffer;
if spike processing pipeline is active **then**
// Do nothing as subsequent spikes will be processed sequentially from the input spike buffer at the end of `_dma_complete_callback`
else
Trigger *user* event leading to `user_callback` and activate of spike processing pipeline;
end if

Algorithm 3 `user_callback`

```
// Activate spike processing pipeline
for First spike in input spike buffer do
    Use source neuron ID as key for master population table, to locate row in Address list
    if Synaptic Matrix is stored in SDRAM then
        DMA synaptic row from synaptic matrix in SDRAM;
    else
        Extract synaptic row from local synaptic matrix in DTCM;
    end if
end for
```

Algorithm 4 `_dma_complete_callback`

```
// Process Synaptic Row
if row does not contain plastic data then
    Extract number of static synapses from header;
    for each static synapse do
        Extract from bottom 16 bits: delay d, target neuron index n, and synapse type t_syn;
        Extract weight from top 16 bits;
        Locate ring buffer slot indexed by [n, t_syn, t + d];
        Add new weight contribution, and check for datatype saturation;
    end for
else if row contains plastic data then
    Extract number of plastic synapses from header
    for each plastic synapse do
        Extract from fixed plastic data: delay d, neuron index n, and synapse type t_syn;
        Extract plastic weight from variable plastic data;
        Perform plastic weight update;
        Add new weight contribution, and check for datatype saturation;
        Write updated weight back to DMA buffer for transfer back to SDRAM;
    end for
end if
```

3 EXAMPLE CODE: RANDOM BALANCED NETWORK

```python
import pyNN.spiNNaker as sim

# Initialise simulator
sim.setup(timestep=1)


# Spike input
poisson_spike_source = sim.Population(250, sim.SpikeSourcePoisson(
    rate=50, duration=5000), label='poisson_source')

spike_source_array = sim.Population(250, sim.SpikeSourceArray,
                                    {'spike_times': [1000]},
                                    label='spike_source')


# Neuron Parameters
cell_params_exc = {
    'tau_m': 20.0, 'cm': 1.0, 'v_rest': -65.0, 'v_reset': -65.0,
    'v_thresh': -50.0, 'tau_syn_E': 5.0, 'tau_syn_I': 15.0,
    'tau_refrac': 0.3, 'i_offset': 0}

cell_params_inh = {
    'tau_m': 20.0, 'cm': 1.0, 'v_rest': -65.0, 'v_reset': -65.0,
    'v_thresh': -50.0, 'tau_syn_E': 5.0, 'tau_syn_I': 5.0,
    'tau_refrac': 0.3, 'i_offset': 0}

# Neuronal populations
pop_exc = sim.Population(500, sim.IF_curr_exp(**cell_params_exc),
                         label='excitatory_pop')

pop_inh = sim.Population(125, sim.IF_curr_exp(**cell_params_inh),
                         label='inhibitory_pop')


# Generate random distributions from which to initialise parameters
rng = sim.NumpyRNG(seed=98766987, parallel_safe=True)

# Initialise membrane potentials uniformly between threshold and resting
pop_exc.set(v=sim.RandomDistribution('uniform',
                                     [cell_params_exc['v_reset'],
                                      cell_params_exc['v_thresh']],
                                      rng=rng))

# Distribution from which to allocate delays
delay_distribution = sim.RandomDistribution('uniform', [1, 10], rng=rng)

# Spike input projections
spike_source_projection = sim.Projection(spike_source_array, pop_exc,
    sim.FixedProbabilityConnector(p_connect=0.05),
    synapse_type=sim.StaticSynapse(weight=0.1, delay=delay_distribution),
    receptor_type='excitatory')

# Poisson source projections
```

```
poisson_projection_exc = sim.Projection(poisson_spike_source, pop_exc,
    sim.FixedProbabilityConnector(p_connect=0.2),
    synapse_type=sim.StaticSynapse(weight=0.06, delay=delay_distribution),
    receptor_type='excitatory')
poisson_projection_inh = sim.Projection(poisson_spike_source, pop_inh,
    sim.FixedProbabilityConnector(p_connect=0.2),
    synapse_type=sim.StaticSynapse(weight=0.03, delay=delay_distribution),
    receptor_type='excitatory')

# Recurrent projections
exc_exc_rec = sim.Projection(pop_exc, pop_exc,
    sim.FixedProbabilityConnector(p_connect=0.1),
    synapse_type=sim.StaticSynapse(weight=0.03, delay=delay_distribution),
    receptor_type='excitatory')
exc_exc_one_to_one_rec = sim.Projection(pop_exc, pop_exc,
    sim.OneToOneConnector(),
    synapse_type=sim.StaticSynapse(weight=0.03, delay=delay_distribution),
    receptor_type='excitatory')
inh_inh_rec = sim.Projection(pop_inh, pop_inh,
    sim.FixedProbabilityConnector(p_connect=0.1),
    synapse_type=sim.StaticSynapse(weight=0.03, delay=delay_distribution),
    receptor_type='inhibitory')

# Projections between neuronal populations
exc_to_inh = sim.Projection(pop_exc, pop_inh,
    sim.FixedProbabilityConnector(p_connect=0.2),
    synapse_type=sim.StaticSynapse(weight=0.06, delay=delay_distribution),
    receptor_type='excitatory')
inh_to_exc = sim.Projection(pop_inh, pop_exc,
    sim.FixedProbabilityConnector(p_connect=0.2),
    synapse_type=sim.StaticSynapse(weight=0.06, delay=delay_distribution),
    receptor_type='inhibitory')


# Specify output recording
pop_exc.record('all')
pop_inh.record('spikes')


# Run simulation
sim.run(simtime=5000)


# Extract results data
exc_data = pop_exc.get_data('spikes')
inh_data = pop_inh.get_data('spikes')


# Exit simulation
sim.end()
```

Code S 1: Example PyNN script for execution via sPyNNaker
